# Intensive Therapy of the Lower Limbs and the Trunk in Children with Bilateral Spastic Cerebral Palsy: Comparing a Qualitative Functional and a Functional Approach

**DOI:** 10.3390/jcm12124078

**Published:** 2023-06-15

**Authors:** Vanessa van Tittelboom, Lieve Heyrman, Josse De Cat, Patrick Algoet, Nicky Peeters, Ipek Alemdaroğlu-Gürbüz, Frank Plasschaert, Katrin Van Herpe, Guy Molenaers, Nele De Bruyn, Ellen Deschepper, Kaat Desloovere, Patrick Calders, Hilde Feys, Christine Van den Broeck

**Affiliations:** 1 Department of Rehabilitation Sciences and Physiotherapy, Ghent University, 9000 Ghent, Belgium; 2 Department of Rehabilitation Sciences, KU Leuven, 3000 Leuven, Belgium; 3 Belgian Bobath Association (ABBV), 1082 Brussels, Belgium; 4 Department of Physiotherapy and Rehabilitation, Hacettepe University, Ankara 06560, Turkey; 5 Department of Orthopedic Surgery, Ghent University Hospital, 9000 Ghent, Belgium; 6 Rehabilitation Centre for Children and Youth, 2242 Pulderbos, Belgium; 7 Pediatric Orthopedics, Department of Orthopedics, University Hospital Leuven, 3000 Leuven, Belgium; 8 Biostatistics Unit, Department of Public Health and Primary Care, Ghent University, 9000 Ghent, Belgium

**Keywords:** cerebral palsy, intensive therapy, lower limbs, trunk control

## Abstract

Few studies have examined the effect of intensive therapy on gross motor function and trunk control in children with cerebral palsy (CP). This study evaluated the effects of an intensive burst of therapy on the lower limbs and trunk by comparing qualitative functional and functional approaches. This study was designed as a quasi-randomized, controlled, and evaluator-blinded trial. Thirty-six children with bilateral spastic CP (mean age = 8 y 9 mo; Gross Motor Function Classification II and III) were randomized into functional (*n* = 12) and qualitative functional (*n* = 24) groups. The main outcome measures were the Gross Motor Function Measure (GMFM), the Quality Function Measure (QFM), and the Trunk Control Measurement Scale (TCMS). The results revealed significant time-by-approach interaction effects for all QFM attributes and the GMFM’s standing dimension and total score. Post hoc tests showed immediate post-intervention gains with the qualitative functional approach for all QFM attributes, the GMFM’s standing and walking/running/jumping dimension and total score, and the total TCMS score. The qualitative functional approach shows promising results with improvements in movement quality and gross motor function.

## 1. Introduction

Cerebral palsy (CP) is the most common cause of physical disability in childhood, with an estimated pooled prevalence of 2.11 per 1000 live births [1,2]. CP denotes a group of permanent disorders of movement and posture development caused by a brain injury occurring in early life [2]. CP etiology is complex. In a large cohort study in Sweden, the etiology of CP was considered prenatal in 38%, peri-/neonatal in 38%, and unclassified in 24% of cases [3]. The Surveillance of Cerebral Palsy in Europe’s database indicates that post-neonatal CP is rare and occurs in approximately 5% of cases [4]. A recent study by Chopra et al. found that up to one in four patients with CP have an underlying genetic condition [5]. Table A1 lists some potential risk factors for CP. Impairments, such as spasticity, reduced selectivity, and postural stability, have been associated with impaired motor function, such as gait deviations. CP’s motor disorders are often accompanied by disturbances in sensation, perception, cognition, communication, and behavior; epilepsy; and secondary musculoskeletal problems [6]. Although the initial neuropathologic lesion is non-progressive, children with CP may develop a range of secondary conditions that will variably affect their functional abilities [7]. Therefore, individualized therapeutic approaches that aim to provide long-term benefits are required [8,9].

Research on the effects of physiotherapeutic interventions in children with CP has increased greatly, showing a wide diversity of techniques and concepts used in variable intensity [8]. Different therapeutic interventions targeting hand function, gross motor function, and postural control have proven their clinical efficacy. However, the results of interventional studies are variable [8,10,11,12], potentially reflecting heterogeneity in clinical presentations within the group of children with CP, differences in the applied therapies, and methodological flaws, such as small sample sizes and the use of general outcomes that lack sensitivity [13]. The various approaches to treating CP are based on different motor learning theories. One of those approaches is the functional approach [14,15,16], in which the focus of assessment and intervention is functionality. The therapist acts mainly as a supervisor, adapting the environment without actively guiding the child’s performance. Other approaches adopt a more qualitative approach to functional training, such as neurodevelopmental treatment (NDT) or the Bobath concept. The children practice the functional task itself and learn to improve the quality of their performance to perform the task more efficiently [17]. The therapist plays a more active role by guiding the movement and providing feedback on the performance.

Besides the intervention type, therapy intensity might play a considerable role in treatment efficacy. A recent systematic review and meta-analysis showed evidence for the effect of intensive training on hand function in children with CP, including short bursts of highly intensive therapy in camp models [18]. In contrast, studies on the effect of intensive therapy on gross motor function are limited, and their results are more inconclusive [18,19]. Interestingly, two studies conducted in children with unilateral and bilateral spastic CP suggested that combined upper- and lower-extremity training in an intensive protocol may effectively improve both upper- and lower-extremity function [20,21]. Nevertheless, the core of this intervention remains bimanual training associated with postural and lower-extremity demands. While it is known that most children with CP have impaired trunk control [10,22], improvement of trunk control has never been included as a main treatment goal and outcome in clinical trials assessing the effect of intensive therapy.

Therefore, this study aimed to investigate the effect of an intensive therapy program using a camp model on qualitative and quantitative parameters of the lower limbs and trunk in children with bilateral spastic CP. The effects of functional and qualitative functional therapeutic approaches were compared.

## 2. Materials and Methods

### 2.1. Study Design

**Figure A1:**
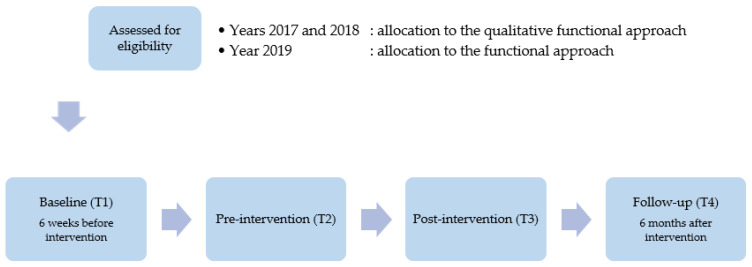
Flowchart of this quasi-randomized trial.

This study was designed as a quasi-randomized, controlled, and evaluator-blinded trial. During three consecutive summers, 36 children (12 per camp) were enrolled in a day-camp model for 10 days. A day-camp model is one in which children are involved in an intensive burst of therapy, involving a certain amount of therapy during the daytime. In the summers of 2017 to 2019, two camps with a qualitative functional approach and one with a functional approach were organized. When they met the eligibility criteria, the children were assigned based on the year of inclusion. Assessments occurred at baseline, pre- and post-intervention, and at a six-month follow-up. The children continued their routine therapy between the baseline and pre-intervention assessments and post-intervention and follow-up assessments. Figure A1 shows a flowchart of this study.

Based on previous research, the sample-size estimate for this study was 12 participants in each group to detect a change of 3% in one of the primary outcome measures, the Gross Motor Function Measure (GMFM), with 80% power [23].

### 2.2. Participants

The eligibility criteria for participants were (1) a diagnosis of bilateral spastic CP, (2) a Gross Motor Function Classification System (GMFCS) level of II or III [24], (3) aged 6–12 years (y), and (4) the ability to understand and follow instructions and complete testing. Children were excluded if they had previously undergone (1) a vertebral fusion, (2) orthopedic surgery or selective dorsal rhizotomy within two years before the start of the study, and (3) if they had received botulinum toxin injections in the lower limb within six months before study enrollment. Children were recruited via CP reference centers, schools for children with motor impairments, and pediatric physical therapists in Belgium. Participants were enrolled between January 2017 and May 2019.

The protocol was performed according to the Helsinki Declaration and approved by the Ethical Committee of the University Hospitals of Ghent and Leuven. Informed consent was obtained from all children’s parents and the children aged 12 and upwards.

### 2.3. Intervention

This study compared two therapeutic approaches. The functional and qualitative functional approaches have specific intrinsic characteristics but also share a common setup and general guidelines. These general and specific guidelines are summarized in Table A2 and Table A3.

#### 2.3.1. Setup and General Guidelines

The intervention setup aimed to provide an intensive burst of physiotherapy focusing on the lower limbs and trunk. Participants were engaged in four-and-a-half hours of treatment per day, five consecutive days a week (Monday to Friday) for two weeks and could sleepover or go home after each camp day. This design resulted in 45 h of therapy, spread across two clusters of five days, with a weekend between both clusters. Pediatric physical therapists and final-year pediatric physical therapy students delivered the therapeutic program. Caregivers and parents of the participants were not involved in the therapeutic program. The main investigators developed the program’s content in collaboration with experienced pediatric physical therapists. Before the camps, information sessions were organized to inform therapists, and a manual was developed that outlined the program’s body, individual goals, and pre-defined exercises. Trained supervisors assisted therapists through the treatment sessions, and an intervision session was organized at the end of each day to ensure compliance with the guidelines.

The therapeutic program’s core comprised five domains: balance, transfers, trunk mobility and trunk stability, walking, and going up and down the stairs. Prerequisites were allocated for each domain (Appendix A, Table A4). Each prerequisite comprised functional exercises with different difficulty levels. The training was organized in individual and group settings. An overview of a daily program can be found in the Appendix A (Table A5).

The motor learning theory was used as one of the foundations for both intervention approaches. Elements such as structure, repetition, and variation are important for enhancing the integration and generalization of motor activities [25]. Individualization of goals, progression, and motivation are also known triggers to reinforce the child’s learning ability. For the latter, each child had a personal form reporting individual impairments and difficulties, steering their therapeutic program. The therapist’s role was to accompany the children through the therapeutic process, giving them an active role in finding solutions to engaging in functional activities. Both approaches also relied on the task-specific model in which treatment involves skill requirements to learn or improve a specific task [25]. Another overarching component of the interventions was the circus theme. Circus activities were selected to optimize engagement in the intervention and ensure high compliance with motor learning principles [26]. In addition, domains were trained using a Nintendo Wii balance board three times a week for 45 min to increase variation and enhance the children’s motivation. A review by Montoro-Cadenas showed that Nintendo Wii therapy could be effective in improving functional and dynamic balance in children with CP, especially when combined with conventional physical therapy [27].

#### 2.3.2. Functional Approach

The functional approach was an activity-focused therapy. Therapists provided an environment that enabled the children to perform self-initiated actions, focusing more on the successful accomplishment of specific tasks rather than the quality of movement. At the beginning of each exercise, children were given cues to generate problem-solving strategies and avoid compensations. In 2020, a group of experts introduced criteria for functional therapy in children with CP [14]. A difference from the functional approach implemented in this study was that goals were formulated by the main investigators, not set in consultation with the children/parents.

#### 2.3.3. Qualitative Functional Approach

The qualitative functional approach was Bobath-concept-oriented and activity- and impairment-focused. The Bobath concept aims to maximize the child’s potential to improve motor competence and prevent secondary musculoskeletal complications [28]. Practice based on the Bobath concept involves an interactive problem-solving approach considering each individual’s clinical presentation and personal goals. Treatment is focused on guiding the individual towards efficient, qualitative movement strategies for task performance [29].

### 2.4. Assessment

Experienced pediatric physical therapists performed the assessments. The primary outcomes were the GMFM-88 [30], the Quality Function Measure (QFM) [31], and the Trunk Control Measurement Scale (TCMS) [32]. The secondary outcomes were the modified Timed Up-and-Go test (mTUG) [33] and the One-Minute Walk Test (1MWT) [34] to measure gait capacity. The assessment tools used have been shown to be reliable and valid [30,31,32,33,34,35]. Baseline assessments were used to determine initial individual treatment objectives for both approaches. Additional assessments at baseline included a clinical examination and gait analysis.

#### 2.4.1. Primary Outcomes

At the body structure and function level, the QFM evaluates key quality attributes of gross motor function in ambulatory children with CP [31]. These attributes are (1) alignment, (2) coordination, (3) weight shift, (4) stability, and (5) dissociated movement. They are assessed using video recordings. Three allocated attributes per item were scored on video using a four-point ordinal scale. The scores for the five attributes were used for analysis. The TCMS assessed static and dynamic sitting balance [32]. This scale comprises three subscales: static sitting balance, selective movement control, and dynamic reaching. The TCMS items were scored in video recordings using a two- or three-point ordinal scale. Both total TCMS and subscale scores were calculated.

At the activity level, the GMFM-88 was used to evaluate gross motor functions in children with CP [30]. The GMFM-88 items were scored in video recordings using a four-point ordinal scale [35]. In this study, children were assessed on dimensions C (kneeling and crawling), D (standing), and E (walking, running, and jumping). Each dimension’s score and a total score (total CDE) were used for analysis.

#### 2.4.2. Secondary Outcomes

At the activity level, gait capacity was assessed using two standardized tests. The mTUG assesses dynamic balance and mobility [33]. The mean score of two attempts was used for analysis. The 1MWT is a validated and user-friendly tool to evaluate walking ability and endurance [34]. The score from one attempt was used for analysis.

#### 2.4.3. Additional Assessments

At the body structure and function level, a clinical examination and three-dimensional gait analysis (3DGA) were performed. Passive and active range of motion and muscle tone of the lower limbs, muscle strength, and selectivity of the lower limbs and the abdominal and back muscles were examined. The 3DGA results included measurement of joint movement during gait (kinematics), moment and power (kinetics), and muscle activity registered by electromyography of both the lower limbs and trunk. The additional assessments were only used to determine initial individual treatment objectives.

### 2.5. Statistics

Both groups were compared at baseline using independent sample *t*-tests. The evolution over time of the primary and secondary outcome measures between the two approaches was investigated using mixed models, adjusting for age and GMFCS level. The analysis was based on a first-order autoregressive covariance structure accounting for the dependencies of observations for a child over time. A Bonferroni correction was applied when comparing the mean outcome measures pairwise between all four time points per camp type. The Bonferroni-adjusted alpha level was 0.008, and corresponding 99.2% confidence intervals are reported. First, interactions between approach and time were analyzed to assess differences in improvement over time between the two treatment approaches. Second, time trends were tested for each treatment approach separately, and pairwise post hoc tests were used to compare individual time points. Estimated marginal means and corresponding confidence intervals were visualized in high–low–close charts. Additionally, subgroup analyses were performed for the GMFCS level. Pre-post effect sizes (ESs) were calculated using Cohen’s *d* formula [36]. According to Cohen, an ES of 0.2–0.5 is considered small, 0.5–0.8 medium, and >0.8 large. Treatment effects were also compared using the minimal detectable change (MDC) and the minimal clinically important difference (MCID) depending on their availability for each outcome measure [31,35,37,38]. Finally, interactions between approach and time were analyzed for a paired sample of both approaches (12 participants per group). Matching was random and based on age and GMFCS level. All statistical analyses were performed in SPSS Statistics for Windows (version 27.0; IBM Corp., Armonk, NY, USA).

## 3. Results

### 3.1. Participants

This study included 36 children. Twenty-four children were enrolled in a qualitative functional camp and twelve in a functional-based camp. Due to botulinum toxin injections or multilevel surgery, eight children in the qualitative functional group and five in the functional group could not be measured at follow-up. The baseline characteristics for both groups are shown in Table 1.

### 3.2. Treatment Efficacy

**Figure 1:**
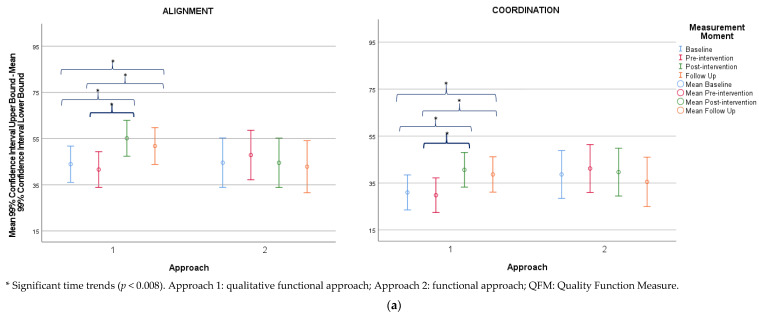

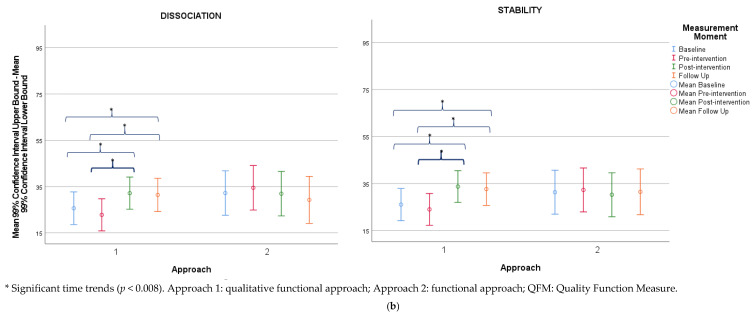

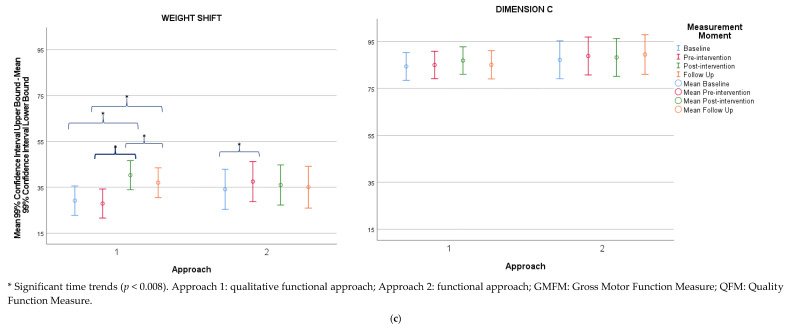

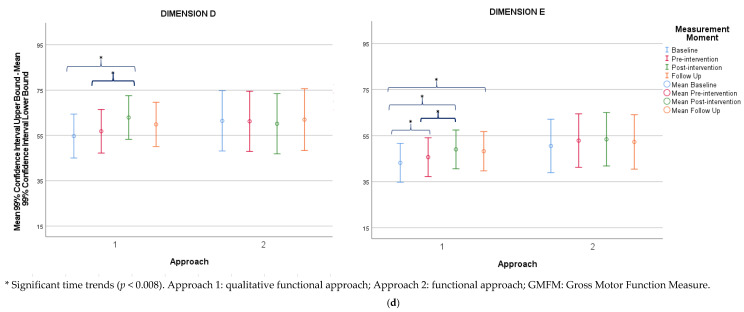

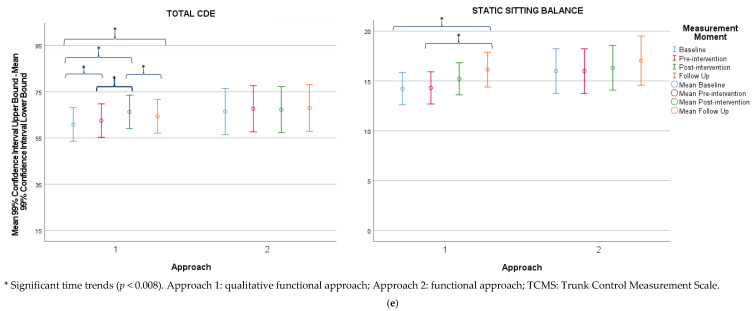

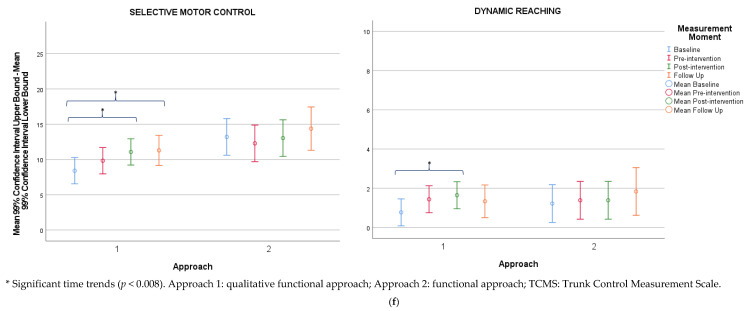

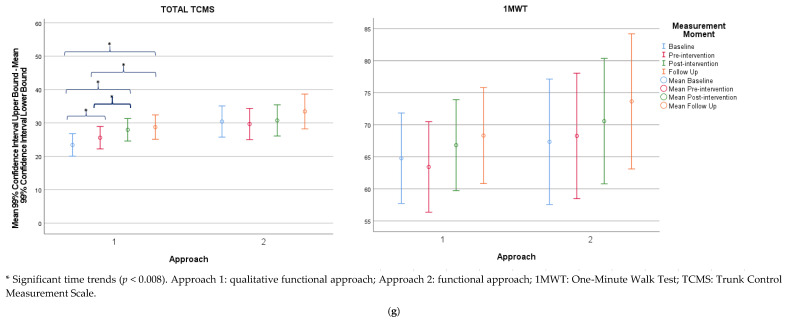

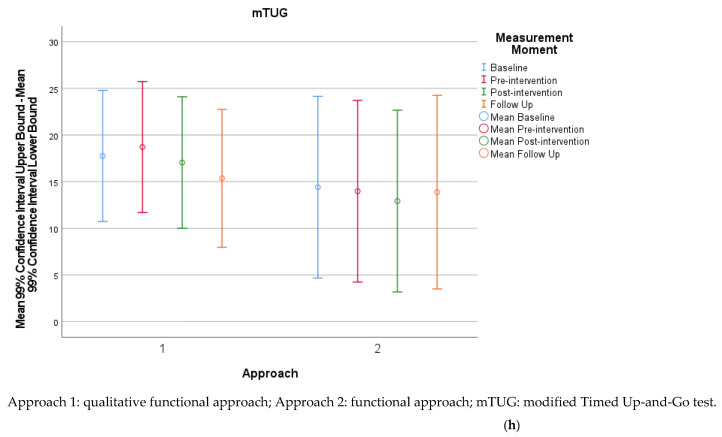
(**a**) High–low charts of the estimated marginal means and corresponding confidence intervals for the alignment and coordination attributes of the QFM. (**b**) High–low charts of the estimated marginal means and corresponding confidence intervals for the dissociation and stability attributes of the QFM. (**c**) High–low charts of the estimated marginal means and corresponding confidence intervals for the weight shift attribute of the QFM and dimension C of the GMFM. (**d**) High–low charts of the estimated marginal means and corresponding confidence intervals for dimensions D and E of the GMFM. (**e**) High–low charts of the estimated marginal means and corresponding confidence intervals for the total CDE score of the GMFM and static sitting balance subscale of the TCMS. (**f**) High–low charts of the estimated marginal means and corresponding confidence intervals for the selective motor control and dynamic reaching subscales of the TCMS. (**g**) High–low charts of the estimated marginal means and corresponding confidence intervals for the total scores of the TCMS and 1MWT. (**h**) High–low charts of the estimated marginal means and corresponding confidence intervals for mTUG outcomes.

**Figure A2:**
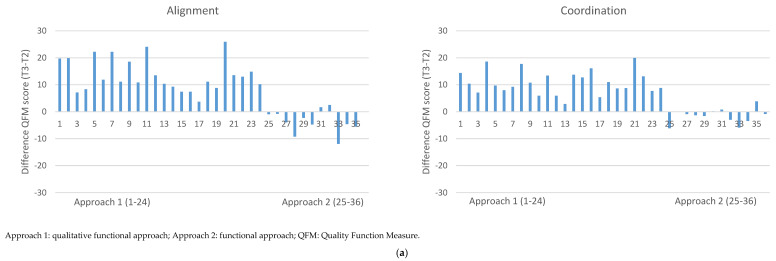

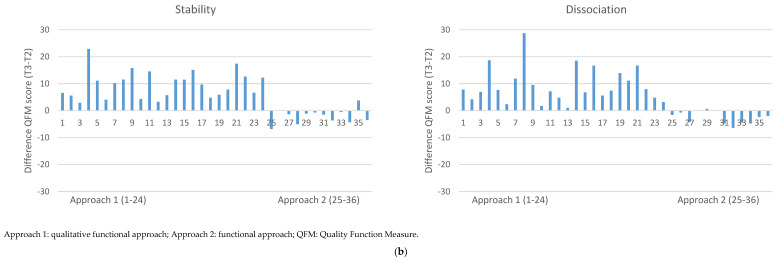

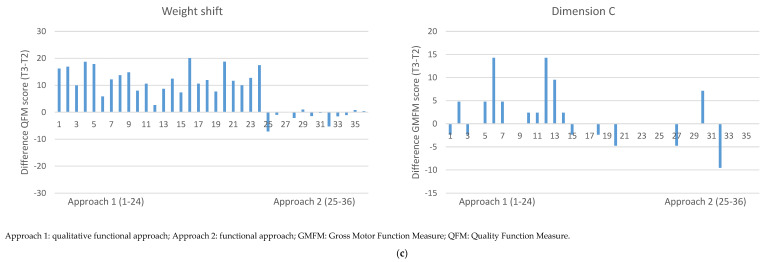

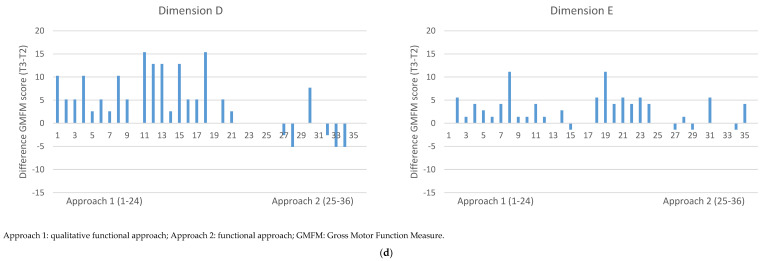

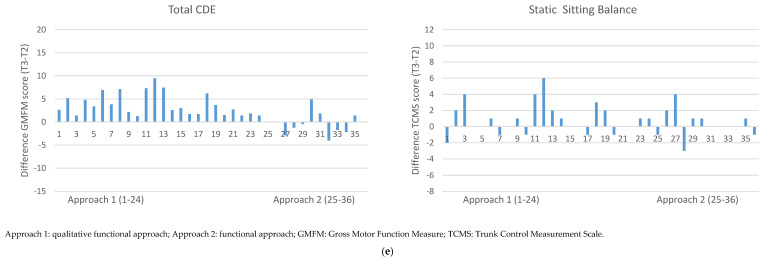

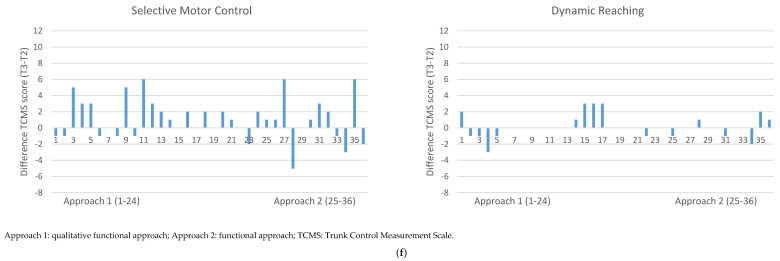

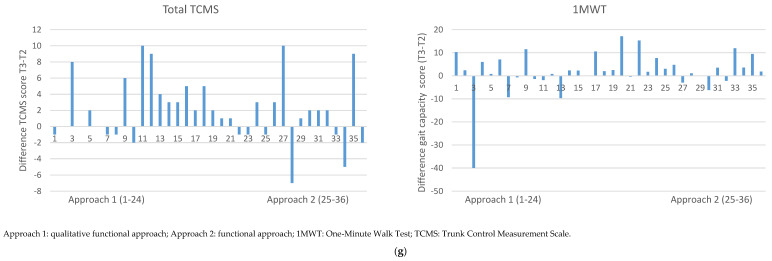

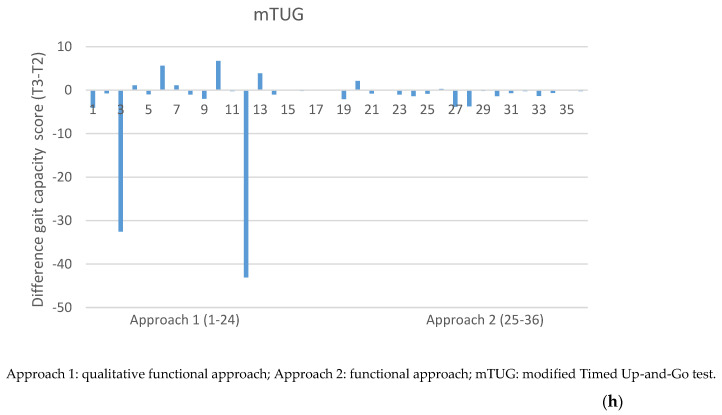
(**a**) Mean changes in pre- to post-intervention scores for both approaches. (**b**) Mean changes in pre- to post-intervention scores for both approaches (continued). (**c**) Mean changes in pre- to post-intervention scores for both approaches (continued). (**d**) Mean changes in pre- to post-intervention scores for both approaches (continued). (**e**) Mean changes in pre- to post-intervention scores for both approaches (continued). (**f**) Mean changes in pre- to post-intervention scores for both approaches (continued). (**g**) Mean changes in pre- to post-intervention scores for both approaches (continued). (**h**) Mean changes in pre- to post-intervention scores for both approaches (continued).

At baseline, all outcome measures did not differ significantly between groups. The results of the outcome measures for both groups are summarized in Table 2, Table 3, Table 4, Table 5, Table 6, Table 7 and Table 8 and in the Appendix A (Table A6 and Table A7). Mean changes between pre- and post-intervention are shown in the Appendix A (Figure A2a–h). Estimated marginal means and corresponding confidence intervals were visualized as high–low–close charts (Figure 1a–h).

#### 3.2.1. Primary Outcomes

The results revealed significant time-by-approach interaction effects for all attributes of the QFM and dimension D and total CDE of the GMFM (Table 2). The qualitative functional approach showed significant time trends for all attributes of the QFM; dimensions D, E, and total CDE of the GMFM; and two subscales (static sitting balance and selective movement control) and total score of the TCMS. No significant time trends were found for the QFM and the GMFM with the functional approach.

Post hoc tests comparing individual time points showed immediate post-intervention gains with the qualitative functional approach for all attributes of the QFM; dimensions D, E, and total CDE of the GMFM; and the total score of the TCMS (Table 3, Table 4 and Table 5). There were no significant changes between post-intervention and follow-up except for weight shift (QFM) and total CDE (GMFM). Post hoc tests showed no significant differences pre- and post-intervention for the functional approach (Table 3, Table 4 and Table 5).

For the qualitative functional approach, the mean pre- to post-intervention differences and standard deviations (SDs) for the five attributes of the QFM ranged from 9.37% to 13.53% and 19.21% to 24.88%, respectively, resulting in medium Ess ranging from 0.49 to 0.63 (Table 7). For the functional approach, the mean pre- to post-intervention differences and SDs for the QFM ranged from −1.47% to −3.34% and 23.51% to 30.51%, respectively, resulting in Ess ranging from −0.06 to −0.11. For the qualitative functional approach, the mean pre- to post-intervention differences and SDs for the GMFM ranged from 1.88% to 6.09% and 15.05% to 27.30%, respectively, resulting in Ess ranging from 0.12 to 0.23, with a small ES for dimension D. For the functional approach, the mean pre- to post-intervention differences and SDs for the GMFM ranged from −1.07% to 0.58% and 13.68% to 32.74%, respectively, resulting in Ess ranging between −0.04 and 0.02. For the qualitative functional approach, the mean pre- to post-intervention differences and SDs for the TCMS ranged from 0.21 to 2.37 units and 1.54 to 9.02 units, respectively, resulting in Ess ranging from 0.14 to 0.28, with small Ess for the subscales static sitting balance, selective motor control, and the total score. For the functional approach, the mean pre- to post-intervention differences and SDs for the TCMS ranged from 0.00 to 1.08 units and 1.38 to 8.59 units, respectively, resulting in Ess ranging from 0.00 to 0.14.

For the qualitative functional approach, the MDC was exceeded for all the attributes of the QFM except stability (Table 8). In contrast, for the functional approach, the MDC was not reached for any primary outcome. The MCID was only exceeded for dimension D of the GMFM with the qualitative functional approach for both GMFCS levels.

#### 3.2.2. Secondary Outcomes

No significant time-by-approach interaction effects were found for the 1MWT or mTUG (Table 6). Time trends within the approaches were not significant.

For the qualitative functional approach, the mean pre- to post-intervention difference and SD for the 1MWT were 3.40 m and 16.16 m, respectively, resulting in a small ES of 0.28 (Table 7). For the functional approach, the mean pre- to post-intervention difference and SD for the 1MWT were 2.31 m and 19.06 m, respectively, resulting in an ES of 0.12. For the qualitative functional approach, the mean pre- to post-intervention difference and SD for the mTUG were −1.66 s and 19.04 s, respectively, resulting in an ES of 0.12. For the functional approach, the mean pre- to post-intervention difference and SD for the mTUG were −1.06 s and 12.76 s, respectively, resulting in an ES of 0.06.

For the qualitative functional approach, the MCID was exceeded for the mTUG for both GMFCS levels (Table 8). For the functional approach, the MCID for the mTUG was exceeded for GMFCS level III.

#### 3.2.3. Subgroup Analysis

For the qualitative approach, the results revealed significant immediate post-intervention gains for all attributes of the QFM and dimensions D, E, and total CDE of the GMFM for GMFCS level II (Appendix A, Table A6, Table A7, Table A8 and Table A9). There were no significant changes between post-intervention and follow-up, except for the weight shift attribute of the QFM. For GMFCS level III, immediate post-intervention gains were found for three attributes of the QFM (alignment, coordination, and weight shift) and dimensions C, D, and total CDE of the GMFM. There were no significant changes between post-intervention and follow-up for dimension D. For the TCMS, gait capacity, and all outcomes of the functional approach, no immediate post-intervention gains were found when analyzing GMFCS levels II and III separately (Appendix A, Table A10, Table A11, Table A12 and Table A13).

#### 3.2.4. Matched-Pair Analysis

When comparing both approaches as two matched groups of 12 participants, the results revealed significant time-by-approach interaction effects for all attributes of the QFM and dimensions C, D, and total CDE of the GMFM (Appendix A, Table A14). The qualitative functional approach showed significant time trends for all attributes of the QFM and dimensions C, D, E, and total CDE of the GMFM. A significant time trend was found in the functional approach for the coordination attribute of the QFM. For both approaches, no significant time trends were found for the TCMS and gait capacity.

## 4. Discussion

A quasi-randomized, controlled trial involving 36 children with bilateral spastic CP was conducted to investigate the effect of an intensive therapy program on qualitative and quantitative parameters of the lower limbs and trunk. A qualitative functional approach and a functional approach were compared. Significant time-by-approach interactions were found for the five attributes of the QFM and dimension D and the total CDE of the GMFM. No significant differences between approaches were found for the TCMS and gait capacity. Time trends within both approaches showed significant improvements for participants with the qualitative functional approach, with significant post-intervention gains for all attributes of the QFM; dimensions D, E, and total CDE of the GMFM; and total TCMS. There were no significant post-intervention gains with the functional approach.

Several interventions in children with CP, such as pharmacological interventions, orthoses, or surgery, and some therapeutic approaches, such as the Bobath concept, aim to improve biomechanical alignment and movement quality during functional activities [39]. Nevertheless, the effectiveness of such therapeutic approaches on functional outcomes has been debated [11,12]. An important emphasis of the qualitative functional approach was facilitating more efficient, qualitative movement strategies during functional activities. Based on the principle of specificity of training, the current results showed that participants enrolled in the qualitative functional approach improved with respect to the QFM outcomes. The same group showed significant improvements for the GMFM. This finding might reflect that the qualitative functional approach generates learning through an active exploration by the children, with the transfer of enhanced motor strategies to function. These results are consistent with a study that examined two different intensities of NDT in children with CP [40]. Their results showed significant improvements in the GMFM outcomes for both intensities, with greater improvements in the more intensive group. However, there were no significant changes between post-intervention and follow-up in this study, except for weight shift and total CDE. Effect sizes for the qualitative functional approach were small to medium for all attributes of the QFM and dimension D of the GMFM. The MDC was reached for four attributes of the QFM and dimension D of the GMFM. These results emphasize the clinical value of the effect of the qualitative functional approach.

Previous research investigated the effect of intensive, activity-focused therapy on movement quality using the Gross Motor Performance Measure (GMPM) [39,41]. Consistent with the outcome of the QFM with the functional approach in this study, the results revealed no significant post-intervention changes. However, one study demonstrated significant improvement in GMPM scores for improved items of the GMFM but not for items that maintained the same GMFM score [39]. The authors concluded that improvement in quality attributes of the GMPM may be a prerequisite for enhanced motor abilities. In this study, Ess were below 0.14 for the QFM and GMFM, and MDCs were not reached with the functional approach. In contrast, previous research found significant improvement in gross motor function after intensive activity-focused therapy, such as Hand and Arm Bimanual Intensive Training Including Lower Extremity (HABIT-ILE) [21,39,41,42]. HABIT-ILE is a bimanual training approach that continuously incorporates lower-extremity function and postural control in children with bilateral spastic CP [21]. Despite the small sample size of the intervention group in that study (*n* = 10), the results for the day-camp model over 13 days showed post-intervention improvements in the GMFM-66. Besides methodological differences (population and intervention), differences in the results between this previous and the current study might be due to the greater responsiveness of the GMFM-66 compared to the GMFM-88 [43]. Another discrepancy with previous research was the goal-setting process. In this study, the goals were not formulated in consultation with parents and/or the child but defined by the main researchers, which may have influenced the effectiveness of the functional approach. However, a study comparing the results of intensive functional therapy using two different goal-setting strategies (broad, generalized aims decided upon by the children’s physiotherapist versus the use of specific, measurable goals set by the interaction between the child’s physiotherapist and the child, parents, and teachers) showed no effect of the type of goal setting on study outcomes [41].

Regarding trunk control, post hoc tests in this study showed a significant time trend for total TCMS in the qualitative functional approach. The absence of significant results for the subscales might have been due to the specificity of the training. Most exercises in the therapeutic program were closed-chain activities in sitting or standing positions. The items in the TCMS are all open-chained tasks. Moreover, the subscales of the TCMS might be less sensitive to change since the score range is smaller. Nevertheless, the results showed small Ess for two subscales (static sitting balance and selective motor control) for the qualitative functional approach. A previous study on 10 children with bilateral spastic CP (2–9 y) compared the effect of task-oriented training and NDT on sitting posture using the GMFM and electromyography [44]. The children improved in both intervention groups. Nevertheless, the sample sizes were small (*n* = 5 for both groups), a clear description of the interventions was lacking, and the outcome measures might not be representative of changes in postural control.

Finally, no significant changes in gait capacity (1MWT and mTUG) were found for both groups in this study. However, a small ES of 0.28 for the qualitative functional approach was found for the 1MWT. The previously mentioned activity-based HABIT-ILE approach has shown significant differences for the 6MWT [21]. It is plausible that more time was dedicated to walking (18% of the intervention time) in the latter study than in this study.

Overall, the smaller sample size of the functional group than the qualitative functional group could also have caused the lack of significant results for this group in this study. Therefore, a matched-pair analysis was conducted with 12 participants for each approach. The results showed no differences in significant time effects favoring the functional approach, except for the coordination attribute of the QFM (*p*-value of 0.007 instead of 0.009). However, post hoc analysis of the entire study group showed that this time effect might reflect a change between follow-up and pre-intervention results rather than immediate pre- to -post-intervention gains. In addition, as mentioned above, the Ess for pre- to post-intervention changes were all below 0.14 for the functional approach. Moreover, MDCs and MCIDs were not reached for any of the outcomes within the functional approach.

We also investigated whether the GMFCS level would influence treatment outcomes. Overall, subgroup analyses showed similar results for both GMFCS levels in this study. Interestingly, a significant post-intervention change was found for dimension C of the GMFM in the qualitative functional group for GMFCS level III. Participants in GMFCS level II often had a maximum-score pre-intervention for dimension C. This ceiling effect might have influenced the non-significant results for the entire group for this dimension. However, this subgroup analysis should be interpreted cautiously due to the small number of participants. The results of a previous study implied that children classified in GMFCS-levels I–II improved more in gross motor function than children classified in levels III–V after an intensive goal-directed, activity-focused intervention [39]. The authors interpreted these results as consistent with the motor developmental curves for CP [40,45]. Each individual’s change in score (pre- to post-intervention difference) was interpreted relative to available data on the MDCs or MCIDs of the clinical measures [34,35,37,38] to identify the variability in the response between participants. Overall, more participants with the qualitative functional approach achieved the MDC or MCID values for the QFM, GMFM, TCMS, and 1MWT. When accounting for age and GMFCS level, children with a higher motor function level (GMFCS level II) and younger children (aged 6–9 y) tended to show improvements in more outcome measures, except for gait capacity, for which we saw the opposite effect. The neuroplasticity phenomenon may partially explain the different responses to the intensive intervention. Previous research indicated the importance of starting early with therapy, given the better opportunities for neuroplasticity in younger children [18]. Intensive training is recommended before age seven because children under this age make the greatest progress when learning new functional skills based on brain maturation and neuroplasticity principles.

## 5. Study Limitations

This study warrants some critical reflections. Due to time limitations, a second activity-based camp could not be organized. Nevertheless, the matched-pair analysis and the mean pre- to post-intervention differences support that sample-size differences may not have greatly impacted the results.

Another limitation is that the functional approach implemented in this study failed to strictly meet all the criteria for functional therapy. Goal setting in this study was the same for both the qualitative functional and functional approaches with individual-, impairment-, and activity-focused goals for each participant set by the therapists.

Future research could involve parents and caregivers, particularly to assess the long-term effects of the approaches. In addition, monitoring the usual care and routines of the children before and after the intensive boost of therapy could be an added value to detect possible influencing factors.

Progression was partially standardized. Variations in the exercises elaborated per the prerequisite of each domain were implemented in the program (e.g., with or without support or with a wide or narrow base of support). Non-standardized variations were adaptations the therapists made to the exercises based on specific abilities or evolutions of the children. The fact that some of the implemented progressions were not standardized and were rather based on the therapist’s expertise reduced the reproducibility of the program. In contrast to a strength training program, a lack of standardization is inherent to programs including treatment approaches, such as the Bobath concept or functional therapy, in which guidelines refer to broader concepts or ideas.

Finally, an important consideration is the feasibility of this type of intensive therapy program. Such programs demand the involvement of many therapists. In Belgium, intensive bursts of therapy are possible in specialized rehabilitation centers after an orthopedic intervention. Since intensive therapy already has shown its efficacy within usual care, such as short bursts of upper-limb training, and considering the needs of parents and children to be offered tailored camps, the social security system should be able to propose adequate financial interventions.

## 6. Conclusions

This study showed greater improvements for the intensive qualitative functional, Bobath-concept-oriented, impairment- and activity-focused approach than for the intensive functional activity-focused approach. Significant post-intervention gains were found for movement quality (QFM) and gross motor function (GMFM). Effects on trunk control (TCMS) were limited, and no significant effects were found for either group on gait capacity (1MWT and mTUG). Subgroup analyses showed similar results for both GMFCS levels II and III. These findings support the importance of interventions focusing on performance quality during functional activities. Future research could implement parental involvement and study the short- and long-term effects of the different approaches in a larger sample.

## Figures and Tables

**Table 1:** Baseline characteristics for both groups.

			Qualitative Functional Approach	Functional Approach	Total Group
Number			24 *	12	36
Age (y and m)		Mean (SD)	8 y 6 m (1.84)	8 y 10 m (2.23)	8 y 7 m (1.96)
Sex					
Female		*n* (%)	14 (58)	3 (25)	17 (47)
Male		*n* (%)	10 (42)	9 (75)	19 (53)
GMFCS level					
II		*n* (%)	15 (62)	7 (58)	22 (61)
III		*n* (%)	9 (37)	5 (42)	14 (39)
Lost to follow-up		*n* (%)	8 (33) II: 6 (6 BoNT-A) III: 2 (1 ML, 1 SDR)	5 (42) II: 2 (1 BoNT-A, 1 ML) III: 3 (2 ML, 1 SDR)	13 (36)

BoNT-A: Botulinum toxin type-A injections; GMFCS: Gross Motor Function Classification System; m: months; ML: multilevel surgery; n: number; SDR: selective dorsal rhizotomy; y: years. * For the qualitative functional approach, four children were excluded for baseline assessments of the QFM and GMFM due to assessment errors.

**Table 2:** Descriptive statistics (means (SDs)) and statistical comparisons (*p*-values) of outcome measures at baseline, pre- and post-intervention, and at the six-month follow-up.

	Approach	Baseline	Pre-Intervention	Post-Intervention	Follow-Up	Time * Approach	Qualitative Functional (1)	Functional (2)
**QFM**								
Alignment	1	54.68 (28.39)	48.57 (25.08)	62.10 (24.68)	54.08 (36.99)	*<0.001*	*<0.001*	0.410
	2	49.68 (28.15)	52.96 (29.48)	49.63 (31.53)	61.53 (31.07)			
Coordination	1	40.15 (21.18)	35.16 (20.48)	45.97 (23.40)	41.88 (24.66)	*<0.001*	*<0.001*	0.009
	2	42.52 (26.18)	45.05 (26.80)	43.51 (26.88)	44.37 (20.60)			
Dissociation	1	33.38 (19.51)	27.46 (17.07)	36.83 (21.37)	34.19 (22.61)	*<0.001*	*<0.001*	0.095
	2	35.46 (22.59)	37.72 (23.76)	35.18 (23.36)	37.71 (19.98)			
Stability	1	34.49 (19.49)	29.09 (18.05)	38.80 (21.73)	35.01 (22.13)	*<0.001*	*<0.001*	0.289
	2	35.10 (23.63)	36.08 (24.62)	34.02 (24.02)	40.00 (20.91)			
Weight shift	1	37.84 (19.77)	32.97 (18.47)	45.33 (20.49)	39.00 (21.43)	*<0.001*	*<0.001*	0.020
	2	37.74 (22.68)	41.09 (24.28)	39.62 (24.18)	44.73 (22.83)			
**GMFM**								
Dimension C	1	89.76 (15.15)	88.10 (16.16)	89.98 (13.95)	86.16 (16.97)	0.108	0.033	0.419
	2	89.29 (14.27)	90.87 (13.35)	90.28 (14.01)	93.20 (12.93)			
Dimension D	1	64.36 (25.75)	62.71 (26.78)	68.80 (25.61)	62.34 (29.51)	*<0.001*	*<0.001*	0.702
	2	65.60 (27.19)	65.38 (28.71)	64.32 (27.65)	71.79 (24.28)			
Dimension E	1	55.14 (26.11)	52.26 (27.01)	55.61 (27.62)	53.21 (30.48)	0.185	*<0.001*	0.116
	2	55.21 (30.80)	57.52 (21.48)	58.10 (32.99)	64.88 (28.53)			
Total CDE	1	69.75 (21.74)	67.69 (22.41)	71.47 (21.59)	67.24 (25.03)	*<0.001*	*<0.001*	0.324
	2	70.03 (23.39)	71.26 (24.04)	70.90 (23.92)	67.62 (21.14)			
**TCMS**								
Static sitting balance	1	14.75 (4.66)	14.83 (4.42)	15.75 (3.78)	16.80 (2.93)	0.769	*0.006*	0.587
	2	16.33 (2.77)	16.33 (3.03)	16.67 (2.50)	17.29 (1.50)			
Selective movement control	1	9.00 (4.76)	10.42 (4.78)	11.67 (4.26)	12.13 (4.42)	0.077	*0.003*	0.290
	2	13.83 (5.57)	12.92 (5.95)	13.67 (5.07)	14.43 (5.50)			
Dynamic reaching	1	0.88 (0.80)	1.54 (1.53)	1.75 (1.73)	1.40 (0.91)	0.423	0.041	0.710
	2	1.33 (1.23)	1.50 (1.51)	1.50 (1.24)	1.86 (2.27)			
Total TCMS	1	24.63 (8.95)	26.79 (9.43)	29.17 (8.26)	30.33 (7.44)	0.780	*<0.001*	0.162
	2	31.50 (9.07)	30.75 (9.39)	31.83 (7.78)	33.57 (8.70)			
**Gait Capacity**								
1MWT	1	67.72 (20.10)	66.37 (15.85)	70.85 (16.48)	72.46 (16.64)	0.679	0.043	0.272
	2	69.96 (17.01)	70.88 (18.62)	73.19 (19.50)	81.02 (13.51)			
mTUG	1	15.77 (17.32)	16.73 (22.76)	14.40 (15.33)	15.05 (15.21)	0.680	0.286	0.885
	2	13.07 (12.08)	12.64 (12.75)	11.58 (12.76)	9.10 (4.70)			

Significant time effects (*p* < 0.008) are shown in italics. *: by; Approach 1: qualitative functional approach; Approach 2: functional approach; 1MWT: One-Minute Walk Test; GMFM: Gross Motor Function Measure; mTUG: modified Timed Up-and-Go test; QFM: Quality Function Measure; TCMS: Trunk Control Measurement Scale.

**Table 3:** Pairwise post hoc tests comparing individual time points within each approach for the QFM.

			Alignment				Coordination				Dissociation				Stability				Weight Shift			
Approach	Time A	Time B	Mean Difference (A–B)	Significance	Lower Bound	Upper Bound	Mean Difference (A–B)	Significance	Lower Bound	Upper Bound	Mean Difference (A–B)	Significance	Lower Bound	Upper Bound	Mean Difference (A–B)	Significance	Lower Bound	Upper Bound	Mean Difference (A–B)	Significance	Lower Bound	Upper Bound
**Qualitative functional**	**2**	**1**	−2.30	0.069	−5.76	1.07	−1.20	0.223	−3.73	1.40	−2.84	0.018	−6.03	0.35	−2.05	0.035	−4.64	0.54	−1.24	0.167	−3.63	1.16
	**3**	**1**	11.23	*<0.001*	6.78	15.69	9.65	*<0.001*	6.23	13.07	6.53	*<0.001*	2.32	10.74	7.67	*<0.001*	4.23	11.11	11.13	*<0.001*	7.95	14.31
		**2**	13.53	*<0.001*	10.45	16.62	10.81	*<0.001*	8.47	13.16	9.37	*<0.001*	6.45	12.29	9.72	*<0.001*	7.35	12.09	12.36	*<0.001*	10.18	14.55
	**4**	**1**	7.87	*<0.001*	2.21	13.53	7.68	*<0.001*	3.30	12.05	5.77	*0.004*	0.44	11.11	6.59	*<0.001*	2.20	10.98	7.86	*<0.001*	3.81	11.92
		**2**	10.17	*<0.001*	5.42	14.92	8.85	*<0.001*	5.20	12.49	8.62	*<0.001*	4.13	13.10	8.64	*<0.001*	4.97	12.31	9.10	*<0.001*	5.71	12.49
		**3**	−3.36	0.017	−7.11	038	−2.00	0.067	−4.83	0.89	−0.75	0.570	−4.30	2.80	−1.10	0.314	−3.96	1.80	−3.26	*0.001*	−5.93	−0.60
**Functional**	**2**	**1**	3.28	0.045	−1.08	7.65	2.53	0.043	−0.79	5.85	2.27	0.142	−1.87	6.40	1.00	0.435	−2.38	4.33	3.35	*0.004*	0.26	6.45
	**3**	**1**	−0.06	0.980	−6.06	5.95	1.00	0.563	−3.62	5.61	−0.28	0.896	−5.95	5.40	−1.10	0.533	−5.72	3.56	1.88	0.240	−2.41	6.17
		**2**	−3.34	0.042	−7.07	1.03	−1.50	0.215	−4.86	1.78	−2.54	0.100	−6.68	1.59	−2.10	0.102	−5.40	1.30	−1.47	0.203	−4.57	1.62
	**4**	**1**	−1.69	0.571	−9.70	6.32	−3.20	0.174	−9.36	3.04	−2.96	0.295	−10.51	4.59	0.20	0.938	−6.04	6.40	0.91	0.670	−4.83	6.66
		**2**	−4.97	0.059	−11.97	2.02	−5.69	*0.005*	−11.06	−0.32	−5.22	0.036	−11.83	1.38	−0.80	0.693	−6.20	4.61	−2.44	0.192	−7.43	2.56
		**3**	−1.64	0.437	−7.29	4.02	−4.15	0.011	-8.47	0.16	−2.68	0.180	−8.03	2.67	1.30	0.438	-3.09	5.61	−0.97	0.518	−4.99	3.05

Significant time trends (*p* < 0.008) are shown in italics. Time 1: baseline; Time 2: pre-intervention; Time 3: post-intervention; Time 4: six-month follow-up; QFM: Quality Function Measure.

**Table 4:** Pairwise post hoc tests comparing individual time points within each approach for the GMFM.

			Dimension C				Dimension D				Dimension E				Total CDE			
Approach	Time A	Time B	Mean Difference (A–B)	Significance	Lower Bound	Upper Bound	Mean Difference (A–B)	Significance	Lower Bound	Upper Bound	Mean Difference (A–B)	Significance	Lower Bound	Upper Bound	Mean Difference (A–B)	Significance	Lower Bound	Upper Bound
**Qualitative functional**	**2**	**1**	0.63	0.481	−1.76	3.02	2.12	0.066	−0.95	5.20	2.46	*0.003*	0.31	4.61	1.71	*0.003*	0.18	3.25
	**3**	**1**	2.51	0.035	−0.66	5.68	8.21	*<0.001*	4.10	12.31	5.82	*<0.001*	2.93	8.71	5.49	*<0.001*	3.43	7.55
		**2**	1.88	0.022	0.63	4.07	6.09	*<0.001*	3.28	8.90	3.35	*<0.001*	1.40	5.32	3.77	*<0.001*	2.38	5.18
	**4**	**1**	0.69	0.648	−3.35	4.72	5.15	0.010	−0.12	10.42	5.01	*<0.001*	1.30	8.73	3.60	*<0.001*	0.94	6.26
		**2**	0.06	0.963	−3.32	3.44	3.03	0.066	−1.36	7.41	2.55	0.028	−0.53	5.63	1.89	0.023	−0.31	4.09
		**3**	−1.83	0.067	−4.49	0.83	−3.06	0.018	−6.49	0.36	−0.80	0.369	−3.20	1.60	−1.89	*0.004*	−3.60	−0.18
**Functional**	**2**	**1**	1.59	0.170	−1.51	4.68	−0.21	0.885	−4.19	3.76	2.31	0.027	−0.47	5.10	1.20	0.097	−0.75	3.21
	**3**	**1**	0.99	0.533	−3.28	5.26	−1.28	0.535	−6.82	4.26	2.89	0.048	−1.00	6.79	0.90	0.403	−1.91	3.65
		**2**	−0.60	0.605	−3.69	2.50	−1.07	0.471	−5.05	2.91	0.58	0.576	−2.20	3.46	−0.40	0.624	−2.34	1.62
	**4**	**1**	2.22	0.298	−3.49	7.93	0.54	0.846	−6.92	8.00	1.75	0.374	−3.52	7.02	1.50	0.277	−2.24	5.30
		**2**	0.63	0.732	−4.34	5.61	0.75	0.754	−5.70	7.21	−0.57	0.737	−5.11	3.97	0.30	0.802	−2.94	3.54
		**3**	1.23	0.411	−2.78	5.24	1.82	0.345	−3.45	7.00	−1.15	0.396	−4.77	2.48	0.70	0.491	−1.92	3.25

Significant time trends (*p* < 0.008) are shown in italics. Time 1: baseline; Time 2: pre-intervention; Time 3: post-intervention; Time 4: six-month follow-up; GMFM: Gross Motor Function Measure.

**Table 5:** Pairwise post hoc tests comparing individual time points within each approach for the TCMS.

			Static Sitting Balance				Selective Motor Control				Dynamic Reaching				Total TCMS			
Approach	Time A	Time B	Mean Difference (A–B)	Significance	Lower Bound	Upper Bound	Mean Difference (A–B)	Significance	Lower Bound	Upper Bound	Mean Difference (A–B)	Significance	Lower Bound	Upper Bound	Mean Difference (A–B)	Significance	Lower Bound	Upper Bound
**Qualitative functional**	**2**	**1**	0.08	0.810	−0.85	1.01	1.42	0.013	−0.09	2.93	0.67	0.017	−0.07	1.41	2.17	*0.006*	0.10	4.24
	**3**	**1**	1.00	0.033	−0.25	2.25	2.67	*<0.001*	0.75	4.58	0.87	*0.007*	0.02	1.73	4.54	*<0.001*	1.79	7.29
		**2**	0.92	0.009	0.01	1.85	1.25	0.028	−0.26	2.76	0.21	0.448	−0.53	0.95	2.37	*0.003*	0.30	4.45
	**4**	**1**	1.92	*0.002*	0.32	3.52	2.89	*0.001*	0.52	5.26	0.56	0.141	−0.45	1.58	5.34	*<0.001*	1.83	8.86
		**2**	1.83	*0.001*	0.41	3.26	1.48	0.072	−0.71	3.66	−0.11	0.774	−1.08	0.87	3.18	*0.008*	0.04	6.31
		**3**	0.92	0.035	−0.24	2.07	0.23	0.742	−1.61	2.06	−0.31	0.337	−1.19	0.56	0.80	0.402	−1.76	3.36
**Functional**	**2**	**1**	0.00	1.000	−1.32	1.32	−0.92	0.249	−3.05	1.22	0.17	0.667	−0.88	1.00	−0.80	0.492	−3.68	2.18
	**3**	**1**	0.33	0.612	−1.43	2.10	−0.17	0.869	−2.87	2.54	0.17	0.713	−1.05	1.38	0.33	0.818	−3.56	4.22
		**2**	0.33	0.497	−1.98	1.65	0.75	0.346	−1.38	2.99	0.00	1.000	−1.04	1.04	1.08	0.322	−1.85	4.01
	**4**	**1**	1.05	0.222	−1.25	3.36	1.16	0.364	−2.25	4.58	0.61	0.265	−0.85	2.08	3.01	0.113	−2.05	8.07
		**2**	1.05	0.171	−1.00	3.11	2.08	0.080	−1.08	5.23	0.45	0.400	−1.97	1.86	3.76	0.028	−0.78	8.29
		**3**	0.72	0.253	−0.97	2.41	1.33	0.185	−1.35	4.01	0.45	0.348	−0.83	1.72	2.68	0.057	−1.07	6.42

Significant time trends (*p* < 0.008) are shown in italics. Time 1: baseline; Time 2: pre-intervention; Time 3: post-intervention; Time 4: six-month follow-up; TCMS: Trunk Control Measurement Scale.

**Table 6:** Pairwise post hoc tests comparing individual time points within each approach for gait capacity.

			1MWT				mTUG			
Approach	Time A	Time B	Mean Difference (A–B)	Significance	Lower Bound	Upper Bound	Mean Difference (A–B)	Significance	Lower Bound	Upper Bound
**Qualitative functional**	**2**	**1**	−1.35	0.299	−4.85	2.14	0.96	0.418	−2.21	4.12
	**3**	**1**	2.05	0.252	−2.74	6.84	−0.71	0.666	−5.08	3.67
		**2**	3.40	0.012	−0.16	6.97	−1.66	0.170	−4.89	1.57
	**4**	**1**	3.57	0.124	−2.61	9.75	−2.38	0.264	−8.06	3.31
		**2**	4.92	0.017	−0.52	10.36	−3.33	0.074	−8.31	1.64
		**3**	1.52	0.305	−2.84	5.88	−1.67	0.258	−5.63	2.29
**Functional**	**2**	**1**	0.91	0.619	−4.03	5.86	−0.43	0.795	−4.91	4.05
	**3**	**1**	3.22	0.199	−3.48	9.93	−1.49	0.515	−7.61	4.64
		**2**	2.31	0.211	−2.63	7.25	−1.06	0.527	−5.54	3.43
	**4**	**1**	6.32	0.058	−2.53	15.18	−0.54	0.858	−8.68	7.59
		**2**	5.41	0.065	−2.40	13.22	−0.11	0.967	−7.25	7.03
		**3**	3.10	0.193	−3.27	9.47	0.95	0.661	−4.84	6.73

Time 1: baseline; Time 2: pre-intervention; Time 3: post-intervention; Time 4: six-month follow-up; 1MWT: One-Minute Walk Test; mTUG: modified Timed Up-and-Go test.

**Table 7:** Pre- and post-intervention effect sizes.

**QFM**					
	Alignment	Coordination	Dissociation	Stability	Weight shift
Approach 1	*0.54*	*0.49*	*0.49*	*0.49*	*0.63*
Approach 2	−0.11	−0.06	−0.11	−0.08	−0.06
**GMFM**					
	Dimension C	Dimension D	Dimension E	Total CDE	
Approach 1	0.13	*0.23*	0.12	0.17	
Approach 2	−0.04	−0.04	0.02	−0.02	
**TCMS**					
	Static sitting balance	Selective motor control	Dynamic reaching	Total TCMS	
Approach 1	*0.22*	*0.28*	0.14	*0.26*	
Approach 2	0.12	0.14	0.00	0.13	
**1MWT**			**mTUG**		
Approach 1	*0.28*		0.12		
Approach 2	0.12		0.06		

Small (0.2–0.5) and medium (0.5–0.8) effect sizes are shown in italics. Approach 1: qualitative functional approach; Approach 2: functional approach; 1MWT: One-Minute Walk Test; GMFM: Gross Motor Function Measure; mTUG: modified Timed Up-and-Go test; QFM: Quality Function Measure; TCMS: Trunk Control Measurement Scale.

**Table 8:** Minimal detectable changes (MDCs), minimal clinically important differences (MCIDs), and mean pre- and post-intervention differences.

			Qualitative Functional Approach	Functional Approach
	MCD	MCID	Mean Difference	Mean Difference
**QFM (%)**				
Alignment	13.5	/	*13.5*	−3.3
Coordination	8.7	/	*10.8*	−1.5
Dissociation	8.4	/	*9.40*	−2.5
Stability	9.9	/	9.70	−2.0
Weight shift	8.4	/	*12.60*	−1.5
**GMFM (%)**				
Dimension C	7.90	/	1.88	−0.59
Dimension D	7.23		6.09	−1.07
II		3.3	*5.46*	−1.10
III		4.1	*6.83*	−1.03
Dimension E	4.16		3.36	0.58
II		4.5	4.26	0.99
III		3.4	1.85	0.00
**TCMS**				
Total TCMS	6	/	2.38	1.08
**Gait capacity**				
1MWT (m)	/			
II		5.1	3.8	2.7
III		3.8	2.7	1.7
mTUG (s)	/			
II		0.7	*0.8*	0.4
III		1.2	*3.3*	2.0

Meaningful differences are shown in italics and green. II: GMFCS level II; III: GMFCS level III; 1MWT: One-Minute Walk Test; m: meter; GMFM: Gross Motor Function Measure; mTUG: modified Timed-Up-and-Go test; s: seconds; QFM: Quality Function Measure; TCMS: Trunk Control Measurement Scale.

## Data Availability

The raw data supporting the conclusions of this article will be made available by the authors on demand.

## Appendix A

**Table A1:** Some potential risk factors for cerebral palsy [46,47,48].

Prenatal risk factors	▪ Maternal factors: TORCH infection (toxoplasmosis, other (syphilis, varicella-zoster, parvovirus B19), rubella, cytomegalovirus, herpes simplex virus), maternal hypothyroidism, iodine deficiency, thrombotic disorders (including factor V Leiden), chorioamnionitis ▪ Fetal factors: teratogen exposure, genetic and metabolic disorders, multiple births, prematurity ▪ Social factors: low socio-economic status
Perinatal risk factors	▪ Birth asphyxia and trauma, non-vertex presentation, placental abruption, rupture of the uterus, prolonged or obstructed labour, post-maturity
Postnatal risk factors	▪ Hyperbilirubinaemia, neonatal sepsis, respiratory distress, early-onset meningitis, intraventricular haemorrhage, post-neonatal head injuries

**Table A2:** General guidelines for the qualitative functional and functional approaches.

Motor Learning Principle	Description
Structure	The therapeutic program had a clear structure with a top-down system: five domains, prerequisites, and exercises.
Repetition	Six individual sessions for each domain were incorporated into the program. Individual logbooks ensured the repetition of the exercises.
Progression	The exercises were progressively graded toward increasing the participants’ capacities. Individual logbooks ensured shaping.
Motivation	Motivation was an important factor during the assessments and therapy. Fun was a key feature and was achieved through rewards, group sessions, attractive leisure moments, and the overarching circus theme.
Individual adjustments	Individual goals were based on baseline assessments. Each participant had their own logbook to ensure the continuity of the therapy.
Therapists guide the execution	The child was considered an active actor seeking problem-solving strategies guided by the therapist.
Focus on function	Functional activities took precedence during at least 70% of the therapeutic sessions.

**Table A3:** Specific guidelines for the qualitative functional and functional approaches.

Qualitative Functional Approach (Pictures a–d)		Functional Approach (Pictures e–h)	
Bobath-concept-oriented		Functional-therapy-oriented	
Activity- and impairment-focused		Activity-focused	
Active and passive preparation of the function - Movement analysis - Biomechanical principles to enhance efficient movement patterns *Therapists identify impairments (clinical reasoning) related to the performance of a task and create more optimal conditions before training for the specific task. The sensory cues and the stimulation of muscles provide input that should enhance active movement*		No preparation of the function	
Hands-on, facilitation through handling skills, and hands-off *Using key points of control to facilitate more efficient, qualitative strategies during the performance of a task*		Hands-off adjustment of the environment	
Verbal cues enhance the quality of movement		No verbal cues on the quality of movement during the performance	
Goal setting on impairment and activity level of the International Classification of Functioning, Disability and Health (ICF) (e.g., enhance the extension of the hip in stance and during sit-to-stand or improve going up and down the stairs holding one handrail)		Goal setting on the activity level of the ICF (e.g., sit-to-stand without using the hands or improve transfers from sitting on the ground to standing position)	
a.	b.	e.	f.

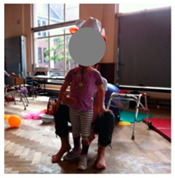
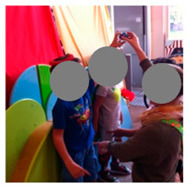
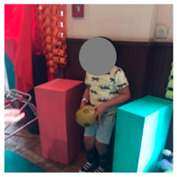
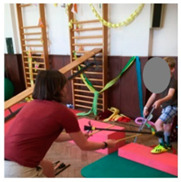
			
Qualitative functional approach		Functional approach	
c.	d.	g.	h.

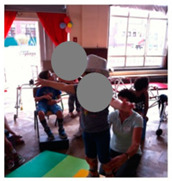
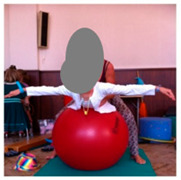
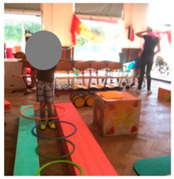
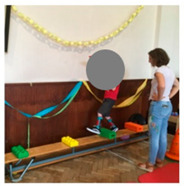
			
Qualitative functional approach		Functional approach	

**Table A4:** Prerequisites per domain.

**Balance**	
Maintaining balance in different postures Balance in stance with one foot in front (length of one step) Dynamic balance	
**Transfers**	
Sit to stand From lying down to stand	Rolling from prone to supine and vice versa Roll-over
**Trunk stability**	**Trunk mobility**
Active upright position of the trunk Maintaining trunk position Trunk stability while moving, in sitting position Trunk stability while moving, in standing position	Trunk mobility in lying position Trunk mobility in sitting position
**Walking**	
Stance phase stability: flat foot, knee and hip extension, trunk alignment Stance on 1 leg Stance on 1 leg while moving the other leg Clearance of the foot during the swing phase Pre-position of the foot at the end of the swing phase	Step length Strength of the lower-limb muscles Walking (forward, sideways, and backwards) Transfers (sitting to standing and vice versa) Walking speed
**Going up and down the stairs**	
Balance in stance Balance on one leg Stance on a step on one leg, the other leg moves Stance with one foot on a step Pushing up on step	Going up and down the stairs: alternating versus not alternating Jumping from a step Active hip- and knee-extension of the stance leg

**Table A5:** Overview of a daily program.

8:30	a.m.	–	9:00	a.m.	Briefing therapists
9:00	a.m.	–	9:45	a.m.	**Group session 1** (warming up with, e.g., stretching and strengthening)
9:45	a.m.	–	10:30	a.m.	**Individual session 1**
10:30	a.m.	–	11:00	a.m.	Break
11:00	a.m.	–	11:45	a.m.	**Individual session 2**
11:45	a.m.	–	12:45	p.m.	Lunch break
12:45	p.m.	–	1:45	p.m.	Standing device group A
1:45	p.m.	–	2:30	p.m.	**Individual session 3**
2:30	p.m.	–	3:00	p.m.	Break
3:00	p.m.	–	3:45	p.m.	**Individual session 4**
3:45	p.m.	–	4:00	p.m.	Break
4:00	p.m.	–	4:30	p.m.	**Group session 2** (showing newly achieved skills, preparing the circus show)
4:30	p.m.	–	5:15	p.m.	Briefing therapists
6:30	p.m.	–	7:30	p.m.	Standing device group B

**Table A6:** Subgroup analysis for the qualitative functional approach for the Quality Function Measure (QFM).

			Alignment				Coordination				Dissociation				Stability				Weight Shift			
GMFCS Level	Time A	Time B	Mean Difference (A–B)	Significance	Lower Bound	Upper Bound	Mean Difference (A–B)	Significance	Lower Bound	Upper Bound	Mean Difference (A–B)	Significance	Lower Bound	Upper Bound	Mean Difference (A–B)	Significance	Lower Bound	Upper Bound	Mean Difference (A–B)	Significance	Lower Bound	Upper Bound
**2**	**2**	**1**	−3.75	*0.007*	−7.45	−0.06	−1.64	0.118	−4.44	1.16	−4.74	*<0.001*	−8.08	−1.39	−2.83	*0.007*	−5.60	−0.07	−1.7	0.082	−4.38	0.92
	**3**	**1**	9.29	*<0.001*	4.21	14.38	10.91	*<0.001*	7.01	14.81	7.15	*<0.001*	2.54	11.76	8.98	*<0.001*	5.14	12.82	11.65	*<0.001*	7.98	15.33
		**2**	13.05	*<0.001*	9.36	16.74	12.55	*<0.001*	9.75	15.36	11.88	*<0.001*	8.54	15.23	11.81	*<0.001*	9.04	14.57	13.38	*<0.001*	10.73	16.03
	**4**	**1**	6.88	*0.007*	0.14	13.63	8.074	*<0.001*	2.87	13.28	6.17	*0.008*	0.06	12.28	6.97	*<0.001*	1.85	12.09	8.11	*<0.001*	3.22	13.00
		**2**	10.64	*<0.001*	4.76	16.51	9.71	*<0.001*	5.21	14.22	10.91	*<0.001*	5.59	16.23	9.80	*<0.001*	5.37	14.23	9.84	*<0.001*	5.60	14.08
		**3**	−2.41	0.172	−7.13	2.31	−2.84	0.036	−6.43	0.76	−1.0	0.540	−5.25	3.30	−2.0	0.130	−5.55	1.54	−3.54	*0.006*	−6.94	−0.15
**3**	**2**	**1**	1.83	0.439	−4.50	8.16	0.05	0.978	−4.77	4.87	2.3	0.273	−3.39	8.08	0.0	0.986	−4.72	4.79	0.0	0.977	−4.50	4.60
	**3**	**1**	16.16	*<0.001*	8.39	23.94	7.97	*<0.001*	2.01	13.93	7.52	*0.005*	0.48	14.56	6.27	*0.005*	0.40	12.13	10.71	*<0.001*	5.10	16.32
		**2**	14.34	*<0.001*	9.57	19.11	7.92	*<0.001*	4.30	11.54	5.17	*0.002*	0.85	9.49	6.23	*<0.001*	2.66	9.81	10.66	*<0.001*	7.24	14.08
	**4**	**1**	11.49	*0.001*	2.28	20.71	7.09	*0.008*	−0.02	14.20	6.99	0.026	−1.35	15.33	6.345	0.016	−0.64	13.33	7.78	*0.002*	1.10	14.46
		**2**	9.67	*<0.001*	2.64	16.69	7.04	*0.001*	1.66	12.42	4.6	0.052	−1.72	11.01	6.32	*0.002*	1.02	11.62	7.73	*<0.001*	2.66	12.80
		**3**	−4.67	0.021	−10.05	0.71	−0.88	0.564	−4.97	3.21	−0.5	0.770	−5.40	4.34	0.1	0.957	−3.95	4.12	−2.94	0.043	−6.80	0.93

Significant time trends (*p* < 0.008) are shown in italics. GMFCS: Gross Motor Function Classification System.

**Table A7:** Subgroup analysis for the functional approach for the Quality Function Measure (QFM).

			Alignment				Coordination				Dissociation				Stability				Weight Shift			
GMFCS Level	Time A	Time B	Mean Difference (A–B)	Significance	Lower Bound	Upper Bound	Mean Difference (A–B)	Significance	Lower Bound	Upper Bound	Mean Difference (A–B)	Significance	Lower Bound	Upper Bound	Mean Difference (A–B)	Significance	Lower Bound	Upper Bound	Mean Difference (A–B)	Significance	Lower Bound	Upper Bound
**2**	**2**	**1**	3.75	0.068	−1.71	9.10	2.74	0.075	−1.37	6.84	3.07	0.095	−1.83	7.96	1.39	0.359	−2.67	5.43	4.61	*0.002*	0.73	8.49
	**3**	**1**	2.37	0.394	−5.08	9.82	1.24	0.559	−4.46	6.95	0.04	0.989	−6.71	6.78	−1.06	0.614	−6.67	4.56	2.95	0.143	−2.43	8.33
		**2**	−1.33	0.510	−6.73	4.08	−1.49	0.329	−5.60	2.61	−3.03	0.099	−7.93	1.87	−2.44	0.108	−6.49	1.61	−1.66	0.251	−5.54	2.22
	**4**	**1**	3.0	0.397	−6.49	12.50	−4.29	0.119	−11.62	3.05	−3.60	0.263	−12.19	5.00	1.07	0.69	−6.13	8.28	3.27	0.205	−3.62	10.15
		**2**	−0.69	0.820	−8.85	7.47	−7.03	*0.003*	−13.28	−0.78	−6.66	0.017	−14.05	0.73	−0.31	0.891	−6.47	5.84	−1.35	0.540	−7.23	4.54
		**3**	0.63	0.788	−5.72	6.98	−5.53	*0.003*	−10.37	−0.70	−3.63	0.092	−9.38	2.12	2.13	0.232	−2.64	6.89	0.32	0.853	−4.25	4.88
**3**	**2**	**1**	2.70	0.258	−3.70	9.10	2.24	0.216	−2.61	7.10	1.15	0.594	−4.64	6.95	0.40	0.823	−4.39	5.19	1.59	0.352	−3.00	6.18
	**3**	**1**	−3.45	0.294	−12.26	5.36	0.65	0.797	−6.11	7.40	−0.71	0.811	−8.69	7.27	−1.11	0.653	−7.76	5.54	0.38	0.872	−5.98	6.74
		**2**	−6.15	0.011	−12.55	0.25	−1.60	0.377	−6.45	3.26	−1.86	0.389	−7.66	3.93	−1.51	0.397	−6.30	3.28	−1.21	0.479	−5.80	3.38
	**4**	**1**	−11.17	0.023	−24.17	1.83	−0.31	0.933	−10.33	9.71	−1.41	0.747	−13.18	10.35	−2.24	0.542	−12.09	7.61	−4.04	0.251	−13.46	5.38
		**2**	−13.87	*0.002*	−25.53	−2.21	−2.56	0.443	−11.49	6.38	−2.57	0.515	−13.12	7.99	−2.64	0.421	−11.43	6.16	−5.63	0.075	−14.05	2.78
		**3**	−7.72	0.039	−17.68	2.24	−0.96	0.734	−8.56	6.64	−0.70	0.834	−9.72	8.32	−1.13	0.686	−8.62	6.36	−4.42	0.100	−11.59	2.75

Significant time trends (*p* < 0.008) are shown in italics. GMFCS: Gross Motor Function Classification System.

**Table A8:** Subgroup analysis for the qualitative functional approach for the Gross Motor Function Measurement (GMFM).

			Dimension C				Dimension D				Dimension E				Total CDE			
GMFCS Level	Time A	Time B	Mean Difference (A–B)	Significance	Lower Bound	Upper Bound	Mean Difference (A–B)	Significance	Lower Bound	Upper Bound	Mean Difference (A–B)	Significance	Lower Bound	Upper Bound	Mean Difference (A–B)	Significance	Lower Bound	Upper Bound
**2**	**2**	**1**	0.48	0.62	−2.10	3.1	1.54	0.24	−1.97	5.05	2.50	*0.006*	0.10	4.90	1.50	0.022	−0.24	3.24
	**3**	**1**	0.79	0.551	−2.78	4.4	7.18	*<0.001*	2.29	12.07	6.76	*<0.001*	3.39	10.13	4.91	*<0.001*	2.47	7.36
		**2**	0.32	0.741	−2.26	2.9	5.64	*<0.001*	2.13	9.15	4.26	*<0.001*	1.86	6.66	3.40	*<0.001*	1.67	5.15
	**4**	**1**	0.86	0.628	−3.89	5.6	3.85	0.116	−2.69	10.39	6.24	*<0.001*	1.72	10.77	3.61	*0.004*	0.32	6.90
		**2**	0.28	0.804	−3.74	4.5	2.31	0.273	−3.34	7.96	3.74	0.011	−0.15	7.63	2.10	0.047	−0.69	4.93
		**3**	0.16	0.958	−3.24	3.4	−3.33	0.049	−7.83	1.17	−0.52	0.652	−3.61	2.57	−1.30	0.12	−3.54	0.94
**3**	**2**	**1**	1.16	0.481	−3.27	5.6	3.85	0.089	−2.20	9.89	2.25	0.147	−1.90	6.40	2.32	0.04	−0.69	5.33
	**3**	**1**	5.66	*0.006*	0.21	11.1	10.68	*<0.001*	3.21	18.16	4.10	0.034	−1.04	9.25	6.71	*<0.001*	2.98	10.45
		**2**	4.50	*<0.001*	1.17	7.8	6.83	*<0.001*	2.31	11.37	1.85	0.111	−1.25	4.96	4.40	*<0.001*	2.15	6.64
	**4**	**1**	1.39	0.565	−5.09	7.9	7.94	0.018	−0.98	16.87	2.91	0.207	−3.26	9.08	4.06	0.017	−0.43	8.55
		**2**	0.23	0.901	−4.70	5.2	4.10	0.105	−2.65	10.85	0.66	0.702	−3.99	5.31	1.74	0.168	−1.63	5.11
		**3**	−4.27	*0.003*	−8.03	−0.5	−2.74	0.153	−7.86	2.38	−1.19	0.363	−4.70	2.32	−2.66	*0.006*	−5.20	−0.11

Significant time trends (*p* < 0.008) are shown in italics. GMFCS: Gross Motor Function Classification System.

**Table A9:** Subgroup analysis for the functional approach for the Gross Motor Function Measurement (GMFM).

			Dimension C				Dimension D				Dimension E				Total CDE			
GMFCS Level	Time A	Time B	Mean Difference (A–B)	Significance	Lower Bound	Upper Bound	Mean Difference (A–B)	Significance	Lower Bound	Upper Bound	Mean Difference (A–B)	Significance	Lower Bound	Upper Bound	Mean Difference (A–B)	Significance	Lower Bound	Upper Bound
**2**	**2**	**1**	0.68	0.628	−3.1	4.5	1.10	0.565	−4.04	6.23	3.77	*0.005*	0.25	7.29	1.85	0.053	−0.70	4.40
	**3**	**1**	−0.68	0.727	−5.90	4.5	0.00	1.000	−7.16	7.16	4.76	0.011	−0.17	9.69	1.36	0.308	−2.22	4.94
		**2**	−1.36	0.334	−5.14	2.4	−1.10	0.565	−6.23	4.04	0.99	0.449	−2.53	4.51	−0.49	0.606	−3.04	2.06
	**4**	**1**	0.74	0.767	−5.95	7.4	2.49	0.469	−6.72	11.70	3.05	0.201	−3.33	9.42	2.11	0.223	−2.53	6.75
		**2**	0.06	0.978	−5.66	5.8	1.39	0.634	−6.45	9.23	−0.72	0.720	−6.12	4.68	0.26	0.857	−3.66	4.18
		**3**	1.42	0.391	−3.02	5.9	2.49	0.270	−3.56	8.54	−1.71	0.269	−5.87	2.44	0.86	0.502	−2.26	3.76
**3**	**2**	**1**	2.86	0.088	−1.61	7.3	−2.05	0.365	−8.13	4.03	0.28	0.858	−3.89	4.44	0.36	0.747	−2.65	3.37
	**3**	**1**	3.33	0.150	−2.85	9.5	−3.08	0.330	−11.55	5.39	0.28	0.898	−5.56	6.11	0.18	0.910	−4.06	4.41
		**2**	0.48	0.774	−3.99	4.9	−1.03	0.650	−7.10	5.05	0.00	1.000	−4.16	4.16	−0.18	0.870	−3.20	2.83
	**4**	**1**	4.35	0.204	−4.80	13.5	−2.99	0.524	−15.57	9.59	0.38	0.908	−8.32	9.07	0.59	0.801	−5.73	6.92
		**2**	1.49	0.625	−6.69	9.7	−0.94	0.822	−12.14	10.26	0.10	0.973	−7.62	7.81	0.23	0.911	−5.37	5.83
		**3**	1.01	0.697	−5.96	8.0	0.09	0.980	−9.44	9.61	0.10	0.968	−6.45	6.64	0.42	0.814	−4.33	5.16

Significant time trends (*p* < 0.008) are shown in italics. GMFCS: Gross Motor Function Classification System.

**Table A10:** Subgroup analysis for the qualitative functional approach for the Trunk Control Measurement Scale (TCMS).

			Static Sitting Balance				Selective Motor Control				Dynamic Reaching				Total TCMS			
GMFCS Level	Time A	Time B	Mean Difference (A–B)	Significance	Lower Bound	Upper Bound	Mean Difference (A–B)	Significance	Lower Bound	Upper Bound	Mean Difference (A–B)	Significance	Lower Bound	Upper Bound	Mean Difference (A–B)	Significance	Lower Bound	Upper Bound
**2**	**2**	**1**	−0.30	0.533	−1.42	0.88	1.73	0.015	−0.16	3.62	0.87	0.012	−0.05	1.8	2.33	0.018	−0.27	4.94
	**3**	**1**	0.30	0.562	−1.21	1.87	2.93	*0.001*	0.54	5.33	1.20	0.003	0.14	2.3	4.47	*0.001*	1.01	7.93
		**2**	0.60	0.163	−0.55	1.75	1.20	0.090	−0.69	3.09	0.33	0.329	−0.58	1.3	2.13	0.030	−0.47	4.74
	**4**	**1**	1.10	0.140	−0.89	3.11	2.99	0.009	0.01	6.99	1.01	0.034	−0.26	2.3	5.02	*0.003*	0.56	9.49
		**2**	1.37	0.040	−0.40	3.15	1.33	0.225	−1.51	4.02	0.15	0.748	−1.08	1.4	2.7	0.073	−1.31	6.68
		**3**	0.80	0.154	−0.68	2.23	0.06	0.947	−2.28	2.40	−0.19	0.651	−1.29	0.9	0.6	0.651	−2.73	3.83
**3**	**2**	**1**	0.70	0.229	−0.82	2.15	0.89	0.328	−1.55	3.33	0.33	0.449	−0.85	1.5	1.9	0.133	−1.47	5.25
	**3**	**1**	2.11	*0.005*	0.12	4.10	2.22	0.056	−0.87	5.31	0.33	0.516	−1.04	1.7	4.67	*0.006*	0.20	9.13
		**2**	1.44	0.010	−0.04	2.93	1.33	0.144	−1.10	3.77	0.00	1.00	−1.18	1.2	2.78	0.028	−0.58	6.14
	**4**	**1**	3.24	*0.001*	0.72	5.76	2.68	0.059	−1.09	6.46	−0.18	0.769	−1.77	1.4	5.83	*0.006*	0.20	11.45
		**2**	2.57	*0.002*	0.35	4.80	1.80	0.167	−1.67	5.36	−0.51	0.377	−2.05	1.0	3.94	0.037	−1.06	8.93
		**3**	1.10	0.093	−0.66	2.92	0.46	0.668	−2.43	3.35	−0.51	0.320	−1.88	0.9	1.2	0.442	−2.88	5.20

Significant time trends (*p* < 0.008) are shown in italics. GMFCS: Gross Motor Function Classification System.

**Table A11:** Subgroup analysis for the functional approach for the Trunk Control Measurement Scale (TCMS).

			Static Sitting Balance				Selective Motor Control				Dynamic Reaching				Total TCMS			
GMFCS Level	Time A	Time B	Mean Difference (A–B)	Significance	Lower Bound	Upper Bound	Mean Difference (A–B)	Significance	Lower Bound	Upper Bound	Mean Difference (A–B)	Significance	Lower Bound	Upper Bound	Mean Difference (A–B)	Significance	Lower Bound	Upper Bound
**2**	**2**	**1**	−0.14	0.820	−1.83	1.54	−1.29	0.213	−4.05	1.48	0.14	0.775	−1.20	1.49	−1.30	0.366	−5.10	2.53
	**3**	**1**	−0.14	0.865	−2.40	2.11	−0.29	0.827	−3.79	3.22	0.00	1.000	−1.56	1.56	−0.40	0.821	−5.49	4.63
		**2**	0.00	1.000	−1.68	1.68	1.00	0.332	−1.76	3.6	−0.14	0.775	−1.49	1.20	0.90	0.546	−2.96	4.67
	**4**	**1**	0.328	0.798	−2.68	3.25	0.54	0.748	−3.92	4.99	0.81	0.255	−1.08	2.69	1.70	0.489	−4.91	8.33
		**2**	0.403	0.666	−2.22	3.07	1.82	0.237	−2.29	5.93	0.66	0.332	−1.16	2.49	3.0	0.178	−2.94	8.94
		**3**	0.43	0.600	−1.75	2.60	0.82	0.530	−2.68	4.32	0.81	0.191	−0.84	2.45	2.10	0.244	−2.77	7.06
**3**	**2**	**1**	0.20	0.787	−1.79	2.19	−0.40	0.742	−3.67	2.87	0.20	0.735	−1.39	1.79	0.00	1.000	−4.51	4.51
	**3**	**1**	1.00	0.316	−1.67	3.67	0.00	1.000	−4.15	4.15	0.40	0.561	−1.44	2.24	1.40	0.531	−4.59	7.39
		**2**	0.80	0.282	−1.19	2.79	0.40	0.742	−2.87	3.67	0.20	0.735	−1.39	1.79	1.40	0.405	−3.11	5.91
	**4**	**1**	2.10	0.106	−1.36	5.57	2.04	0.296	−3.17	7.24	0.37	0.651	−1.83	2.58	4.80	0.099	−2.94	12.54
		**2**	1.90	0.100	−1.18	4.99	2.44	0.176	−2.36	7.23	0.17	0.827	−1.96	2.30	4.80	0.065	−2.12	11.72
		**3**	1.10	0.241	−1.42	3.62	2.04	0.180	−2.02	6.10	−0.03	0.970	−1.94	1.89	3.40	0.111	−2.29	9.09

GMFCS: Gross Motor Function Classification System.

**Table A12:** Subgroup analysis for the qualitative functional approach for gait capacity.

			1MWT				mTUG			
GMFCS Level	Time A	Time B	Mean Difference (A–B)	Significance	Lower Bound	Upper Bound	Mean Difference (A–B)	Significance	Lower Bound	Upper Bound
**2**	**2**	**1**	−3.23	0.046	−7.55	1.08	0.10	0.945	−3.82	4.02
	**3**	**1**	0.59	0.787	−5.27	6.45	−0.66	0.742	−6.03	4.71
		**2**	3.83	0.019	−0.49	8.14	−0.76	0.604	−4.68	3.17
	**4**	**1**	1.93	0.501	−5.75	9.62	−0.85	0.749	−7.93	6.24
		**2**	5.17	0.042	−1.60	11.93	−0.95	0.683	−7.15	5.26
		**3**	1.34	0.511	−4.14	6.82	−0.19	0.92	−5.19	4.81
**3**	**2**	**1**	1.78	0.39	−3.79	7.35	2.38	0.208	−2.68	7.45
	**3**	**1**	4.45	0.128	−3.35	12.24	−0.92	0.729	−8.07	6.22
		**2**	2.67	0.225	−3.22	8.55	−3.31	0.099	−8.66	2.05
	**4**	**1**	6.18	0.094	−3.64	15.99	−4.85	0.153	−13.91	4.21
		**2**	4.39	0.173	−4.22	13.00	−7.23	0.015	−15.14	0.67
		**3**	1.73	0.492	−5.02	8.47	−3.93	0.088	−10.08	2.22

1MWT: One-Minute Walk Test; mTUG: modified Timed Up-and-Go test; GMFCS: Gross Motor Function Classification System.

**Table A13:** Subgroup analysis for the functional approach for gait capacity.

			1MWT				mTUG			
GMFCS Level	Time A	Time B	Mean Difference (A–B)	Significance	Lower Bound	Upper Bound	Mean Difference (A–B)	Significance	Lower Bound	Upper Bound
**2**	**2**	**1**	1.05	0.655	−5.27	7.36	−0.27	0.901	−6.01	5.48
	**3**	**1**	3.78	0.238	−4.79	12.35	−0.67	0.82	−8.53	7.20
		**2**	2.73	0.247	−3.58	9.05	−0.40	0.852	−6.14	5.34
	**4**	**1**	5.52	0.173	−5.28	16.32	0.26	0.944	−9.71	10.23
		**2**	4.47	0.203	−4.92	13.86	0.53	0.869	−8.08	9.14
		**3**	1.74	0.527	−5.65	9.13	0.93	0.712	−5.81	7.66
**3**	**2**	**1**	0.73	0.794	−6.75	8.20	−0.67	0.792	−7.46	6.13
	**3**	**1**	2.45	0.518	−7.70	12.59	−2.64	0.447	−11.94	6.66
		**2**	1.72	0.536	−5.75	9.20	−1.98	0.436	−8.77	4.82
	**4**	**1**	8.52	0.126	−6.32	23.35	−1.81	0.722	−15.48	11.85
		**2**	7.79	0.122	−5.63	21.22	−1.15	0.803	−13.45	11.16
		**3**	6.07	0.160	−5.47	17.61	0.83	0.833	−9.71	11.37

1MWT: One-Minute Walk Test; mTUG: modified Timed Up-and-Go test; GMFCS: Gross Motor Function Classification System.

**Table A14:** Matched-pair analysis.

	Time * Approach	Qualitative Functional Approach	Functional Approach
**Quality Function Measure (QFM)**			
Alignment	*<0.001*	*<0.001*	0.400
Coordination	*<0.001*	*<0.001*	*0.007*
Dissociation	*<0.001*	*<0.001*	0.130
Stability	*<0.001*	*<0.001*	0.209
Weight shift	*<0.001*	*<0.001*	0.150
**Gross Motor Function Measurement (GMFM)**			
Dimension C	*0.008*	*0.003*	0.475
Dimension D	*0.007*	*0.003*	0.635
Dimension E	0.097	*<0.001*	0.087
Total CDE	*<0.001*	*<0.001*	0.303
**Trunk Control Measurement Scale (TCMS)**			
Static sitting balance	0.773	0.189	0.469
Selective movement control	0.230	0.124	0.212
Dynamic reaching	0.234	0.111	0.689
Total TCMS	0.109	0.029	0.109
**Gait Capacity**			
1MWT	0.442	0.146	0.208
mTUG	0.520	0.201	0.926

Significant time trends (*p* < 0.008) are shown in italics. *: by; 1MWT: One-Minute Walk Test; mTUG: modified Timed Up-and-Go test.

## References

[1] Oskoui M., Coutinho F., Dykeman J., Jette N., Pringsheim T. (2013). An update on the prevalence of cerebral palsy: A systematic review and meta-analysis. Dev. Med. Child Neurol..

[2] Cans C., Guillem P., Baille F., Arnaud C., Chalmers J., Cussen G., McManus V., Parkes J., Dolk H. (2000). Surveillance of cerebral palsy in Europe: A collaboration of cerebral palsy surveys and registers. Dev. Med. Child Neurol..

[3] Himmelmann K., Uvebrant P. (2018). The panorama of cerebral palsy in Sweden part XII shows that patterns changed in the birth years 2007–2010. Acta Paediatr..

[4] Germany L., Ehlinger V., Klapouszczak D., Delobel M., Hollody K., Sellier E., De La Cruz J., Alberge C., Genolini C., Arnaud C. (2013). Trends in prevalence and characteristics of post-neonatal cerebral palsy cases: A European registry-based study. Res. Dev. Disabil..

[5] Chopra M., Gable D.L., Love-Nichols J., Tsao A., Rockowitz S., Sliz P., Barkoudah E., Bastianelli L., Coulter D., Davidson E. (2022). Mendelian etiologies identified with whole exome sequencing in cerebral palsy. Ann. Clin. Transl. Neurol..

[6] Rosenbaum P., Paneth N., Leviton A., Goldstein M., Bax M. (2007). A report: The definition and classification of cerebral palsy—April 2006. Dev. Med. Child Neurol..

[7] Smithers-Sheedy H., Badawi N., Blair E., Cans C., Himmelmann K., Krageloh-Mann I., Mcintyre S., Slee J., Uldall P., Watson L. (2014). What constitutes cerebral palsy in the twenty-first century?. Dev. Med. Child Neurol..

[8] Franki I., Desloovere K., De Cat J., Feys H., Molenaers G., Calders P., Vanderstraeten G., Himpens E., Van den Broeck C. (2012). The Evidence-Base for Conceptual Approaches and Additional Therapies Targeting Lower Limb Function in Children with Cerebral Palsy: A Systematic Review Using the International Classification of Functioning, Disability and Health as a Framework. J. Rehabil. Med..

[9] Trabacca A., Vespino T., Di Liddo A., Russo L. (2016). Multidisciplinary rehabilitation for patients with cerebral palsy: Improving long-term care. J. Multidiscip. Health.

[10] Dewar R., Love S., Johnston L.M. (2015). Exercise interventions improve postural control in children with cerebral palsy: A systematic review. Dev. Med. Child Neurol..

[11] Novak I., Mcintyre S., Morgan C., Campbell L., Dark L., Morton N., Stumbles E., Wilson S.A., Goldsmith S. (2013). A systematic review of interventions for children with cerebral palsy: State of the evidence. Dev. Med. Child Neurol..

[12] Novak I., Morgan C., Fahey M., Finch-Edmondson M., Galea C., Hines A., Langdon K., Namara M.M., Paton M.C., Popat H. (2020). State of the Evidence Traffic Lights 2019: Systematic Review of Interventions for Preventing and Treating Children with Cerebral Palsy. Curr. Neurol. Neurosci. Rep..

[13] Anttila H., Autti-Ramo I., Suoranta J., Makela M., Malmivaara A. (2008). Effectiveness of physical therapy interventions for children with cerebral palsy: A systematic review. BMC Pediatr..

[14] Geijen M., Ketelaar M., Sakzewski L., Palisano R., Rameckers E. (2020). Defining Functional Therapy in Research Involving Children with Cerebral Palsy: A Systematic Review. Phys. Occup. Ther. Pediatr..

[15] Ketelaar M., Vermeer A., Hart H., van Petegem-van Beek E., Helders P.J. (2001). Effects of a functional therapy program on motor abilities of children with cerebral palsy. Phys. Ther..

[16] Salem Y., Godwin E.M. (2009). Effects of task-oriented training on mobility function in children with cerebral palsy. NeuroRehabilitation.

[17] Butler C., Darrah J. (2001). Effects of neurodevelopmental treatment (NDT) for cerebral palsy: An AACPDM evidence report. Dev. Med. Child Neurol..

[18] Myrhaug H.T., Ostensjo S., Larun L., Odgaard-Jensen J., Jahnsen R. (2014). Intensive training of motor function and functional skills among young children with cerebral palsy: A systematic review and meta-analysis. BMC Pediatr..

[19] Arpino C., Vescio M.F., De Luca A., Curatolo P. (2010). Efficacy of intensive versus nonintensive physiotherapy in children with cerebral palsy: A meta-analysis. Int. J. Rehabil. Res..

[20] Bleyenheuft Y., Arnould C., Brandao M.B., Bleyenheuft C., Gordon A.M. (2015). Hand and Arm Bimanual Intensive Therapy Including Lower Extremity (HABIT-ILE) in Children With Unilateral Spastic Cerebral Palsy: A Randomized Trial. Neurorehab. Neural Repair.

[21] Bleyenheuft Y., Ebner-Karestinos D., Surana B., Paradis J., Sidiropoulos A., Renders A., Friel K.M., Brandao M., Rameckers E., Gordon A.M. (2017). Intensive upper- and lower-extremity training for children with bilateral cerebral palsy: A quasi-randomized trial. Dev. Med. Child Neurol..

[22] Heyrman L., Desloovere K., Molenaers G., Verheyden G., Klingels K., Monbaliu E., Feys H. (2013). Clinical characteristics of impaired trunk control in children with spastic cerebral palsy. Res. Dev. Disabil..

[23] Franki I., Van den Broeck C., De Cat J., Tijhuis W., Molenaers G., Vanderstraeten G., Desloovere K. (2014). A randomized, single-blind cross-over design evaluating the effectiveness of an individually defined, targeted physical therapy approach in treatment of children with cerebral palsy. Clin. Rehabil..

[24] Palisano R., Rosenbaum P., Walter S., Russell D., Wood E., Galuppi B. (1997). Development and reliability of a system to classify gross motor function in children with cerebral palsy. Dev. Med. Child Neurol..

[25] Toovey R., Bernie C., Harvey A.R., McGinley J.L., Spittle A.J. (2017). Task-specific gross motor skills training for ambulant school-aged children with cerebral palsy: A systematic review. BMJ Paediatr. Open.

[26] Coulston F., Cameron K.L., Spittle A., Sellick K., Toovey R. (2021). Circus activities as a health intervention for children, youth, and adolescents: A scoping review protocol. JBI Evid. Synth..

[27] Montoro-Cardenas D., Cortes-Perez I., Ibancos-Losada M.D.R., Zagalaz-Anula N., Obrero-Gaitan E., Osuna-Perez M.C. (2022). Nintendo((R)) Wii Therapy Improves Upper Extremity Motor Function in Children with Cerebral Palsy: A Systematic Review with Meta-Analysis. Int. J. Environ. Res. Public Health.

[28] Graham J.V., Eustace C., Brock K., Swain E., Irwin-Carruthers S. (2009). The Bobath Concept in Contemporary Clinical Practice. Top. Stroke Rehabil..

[29] Valvano J., Rapport M.J. (2006). Activity-focused motor interventions for infants and young children with neurological conditions. Infant. Young Child.

[30] Ko J., Kim M. (2013). Reliability and responsiveness of the gross motor function measure-88 in children with cerebral palsy. Phys. Ther..

[31] Wright F.V., Rosenbaum P., Fehlings D., Mesterman R., Breuer U., Kim M. (2014). The Quality Function Measure: Reliability and discriminant validity of a new measure of quality of gross motor movement in ambulatory children with cerebral palsy. Dev. Med. Child Neurol..

[32] Heyrman L., Molenaers G., Desloovere K., Verheyden G., De Cat J., Monbaliu E., Feys H. (2011). A clinical tool to measure trunk control in children with cerebral palsy: The Trunk Control Measurement Scale. Res. Dev. Disabil..

[33] Dhote S.N., Khatri P.A., Ganvir S.S. (2012). Reliability of “Modified timed up and go” test in children with cerebral palsy. J. Pediatr. Neurosci..

[34] McDowell B.C., Humphreys L., Kerr C., Stevenson M. (2009). Test-retest reliability of a 1-min walk test in children with bilateral spastic cerebral palsy (BSCP). Gait Posture.

[35] Franki I., Van den Broeck C., De Cat J., Molenaers G., Vanderstraeten G., Desloovere K. (2015). A study of whether video scoring is a reliable option for blinded scoring of the Gross Motor Function Measure-88. Clin. Rehabil..

[36] Cohen J. (1988). Statistical Power Analysis for the Behavioral Sciences.

[37] Hassani S., Krzak J.J., Johnson B., Flanagan A., Gorton G., Bagley A., Ounpuu S., Romness M., Tylkowski C., Oeffinger D. (2014). One-Minute Walk and modified Timed Up and Go tests in children with cerebral palsy: Performance and minimum clinically important differences. Dev. Med. Child Neurol..

[38] Storm F.A., Petrarca M., Beretta E., Strazzer S., Piccinini L., Maghini C., Panzeri D., Corbetta C., Morganti R., Reni G. (2020). Minimum Clinically Important Difference of Gross Motor Function and Gait Endurance in Children with Motor Impairment: A Comparison of Distribution-Based Approaches. Biomed Res. Int..

[39] Sorsdahl A.B., Moe-Nilssen R., Kaale H.K., Rieber J., Strand L.I. (2010). Change in basic motor abilities, quality of movement and everyday activities following intensive, goal-directed, activity-focused physiotherapy in a group setting for children with cerebral palsy. BMC Pediatr..

[40] Tsorlakis N., Evaggelinou C., Grouios G., Tsorbatzoudis C. (2004). Effect of intensive neurodevelopmental treatment in gross motor function of children with cerebral palsy. Dev. Med. Child Neurol..

[41] Bower E., Michell D., Burnett M., Campbell M.J., McLellan D.L. (2001). Randomized controlled trial of physiotherapy in 56 children with cerebral palsy followed for 18 months. Dev. Med. Child Neurol..

[42] Bower E., McLellan D.L., Arney J., Campbell M.J. (1996). A randomised controlled trial of different intensities of physiotherapy and different goal-setting procedures in 44 children with cerebral palsy. Dev. Med. Child Neurol..

[43] Shi W., Wang S.J., Liao Y.G., Yang H., Xu X.J., Shao X.M. (2006). Reliability and validity of the GMFM-66 in 0-to 3-year-old children with cerebral palsy. Am. J. Phys. Med. Rehab..

[44] Choi M., Lee D., Ro H. (2011). Effect of Task-oriented Training and Neurodevelopmental Treatment on the Sitting Posture in Children with Cerebral Palsy. J. Phys. Ther. Sci..

[45] Rosenbaum P.L., Walter S.D., Hanna S.E., Palisano R.J., Russell D.J., Raina P., Wood E., Bartlett D.J., Galuppi B.E. (2002). Prognosis for gross motor function in cerebral palsy: Creation of motor development curves. JAMA.

[46] Graham D., Paget S.P., Wimalasundera N. (2019). Current thinking in the health care management of children with cerebral palsy. Med. J. Aust..

[47] Patel D.R., Neelakantan M., Pandher K., Merrick J. (2020). Cerebral palsy in children: A clinical overview. Transl. Pediatr..

[48] Sadowska M., Sarecka-Hujar B., Kopyta I. (2020). Cerebral Palsy: Current Opinions on Definition, Epidemiology, Risk Factors, Classification and Treatment Options. Neuropsychiatr. Dis. Treat..

